# Transfection with Plasmid-Encoding lncRNA-SLERCC nanoparticle-mediated delivery suppressed tumor progression in renal cell carcinoma

**DOI:** 10.1186/s13046-022-02467-2

**Published:** 2022-08-19

**Authors:** Weipu Mao, Keyi Wang, Wentao Zhang, Shuqiu Chen, Jinbo Xie, Zongtai Zheng, Xue Li, Ning Zhang, Yuanyuan Zhang, Haimin Zhang, Bo Peng, Xudong Yao, Jianping Che, Junhua Zheng, Ming Chen, Wei Li

**Affiliations:** 1 Department of Urology, School of Medicine, Shanghai Tenth People’s Hospital, Tongji University, No. 301, Yanchang Road, Jing’an District, Shanghai, 200072 P. R. China; 2 Department of Urology, Affiliated Zhongda Hospital of Southeast University, No. 87 Dingjiaqiao, Hunan Road, Gulou District, Nanjing, 210009 P. R. China; 3 Department of Pathology, Beijing Chao-Yang Hospital, Capital Medical University, Beijing, P. R. China; 4 Department of Medical Oncology, Dana-Farber Cancer Institute, Harvard Medical School, Boston, MA USA; 5 The Affiliated Suqian First People’s Hospital of Nanjing Medical University, Suqian, China; 6 Institute for Regenerative Medicine, Wake Forest University, Winston-Salem, NC USA; 7 State Key Laboratory of Oncogenes and Related Genes, Department of Urology, School of Medicine, Renji Hospital, Shanghai Jiao Tong University, Shanghai, P. R. China; 8 Department of Urology, School of Medicine, Renji Hospital, Shanghai Jiao Tong University, Shanghai, P.R. China

**Keywords:** lncRNA-SLERCC, Nanoparticles, Renal cell carcinoma, Gene therapy, Tumor suppressor

## Abstract

**Background:**

The accumulating evidence confirms that long non-coding RNAs (lncRNAs) play a critical regulatory role in the progression of renal cell carcinoma (RCC). But, the application of lncRNAs in gene therapy remains scarce. Here, we investigated the efficacy of a delivery system by introducing the plasmid-encoding tumor suppressor lncRNA-SLERCC (SLERCC) in RCC cells.

**Methods:**

We performed lncRNAs expression profiling in paired cancer and normal tissues through microarray and validated in our clinical data and TCGA dataset. The Plasmid-SLERCC@PDA@MUC12 nanoparticles (PSPM-NPs) were tested in vivo and in vitro, including cellular uptake, entry, CCK-8 assay, tumor growth inhibition, histological assessment, and safety evaluations. Furthermore, experiments with nude mice xenografts model were performed to evaluate the therapeutic effect of PSPM-NPs nanotherapeutic system specific to the SLERCC.

**Results:**

We found that the expression of SLERCC was downregulated in RCC tissues, and exogenous upregulation of SLERCC could suppress metastasis of RCC cells. Furthermore, high expression DNMT3A was recruited at the SLERCC promoter, which induced aberrant hypermethylation, eventually leading to downregulation of SLERCC expression in RCC. Mechanistically, SLERCC could directly bind to UPF1 and exert tumor-suppressive effects through the Wnt/β-catenin signaling pathway, thereby inhibiting progression and metastasis in RCC. Subsequently, the PSPM-NPs nanotherapeutic system can effectively inhibit the growth of RCC metastases in vivo.

**Conclusions:**

Our findings suggested that SLERCC is a promising therapeutic target and that plasmid-encapsulated nanomaterials targeting transmembrane metastasis markers may open a new avenue for the treatment in RCC.

**Supplementary Information:**

The online version contains supplementary material available at 10.1186/s13046-022-02467-2.

## Background

Renal cell carcinoma (RCC), a tumor originating in the parenchymal epithelium of the kidney, accounts for more than 90% of cases of renal cancer; among them, 80-90% cases are clear cell renal cell carcinomas (ccRCC) [1]. The incidence and mortality rates associated with RCC are on a global rise, at a rate of 2-3% per decade [2, 3]. According to worldwide statistics, in 2020, there were more than 430,000 new cases of RCC and nearly 180,000 associated deaths [2]. RCC has a high propensity for malignant metastasis and distant metastases reportedly occur in approximately 25-30% of patients initially diagnosed with RCC; during postoperative follow-up, metastatic lesions are found in approximately half of the remaining cases [4]. Therefore, deciphering the molecular mechanisms underlying RCC progression and metastases is of clinical importance [5].

Long non-coding RNAs (lncRNAs), a class of RNA molecules that are localized in the nucleus or cytoplasm, have transcript lengths greater than 200 nt [6, 7]. Initially, lncRNAs were considered as "junk sequences" during gene transcription, however, with the development of high-throughput technologies, lncRNAs have confirmed involvement in chromatin modification, DNA methylation, histone modification, transcriptional interference, and other important gene expression regulatory processes [8, 9]. Accumulating evidence confirms that lncRNAs play a critical role in the development of the RCC [10, 11], and many lncRNAs are aberrantly expressed in RCC cells and are involved in RCC progression and infiltrative metastasis [12–15]. The main mode of action of these lncRNAs in RCC is through interaction with various RNA molecules and proteins in cis- or trans-action, thereby regulating gene expression at the transcriptional, post-transcriptional, and epigenetic levels [16–18].

MUC12 (Mucin 12, Cell Surface Associated) is a glycoprotein highly expressed on the surfaces of a majority of malignant epithelial tumor cells [19]. MUC12 is one of the most strongly expressed tumor surface antigens that promotes epithelial cell protection, adhesion modulation, and cell growth regulation signaling [20]. MUC12 transmembrane mucin has c-terminal tandem repeats, three EGF-like sequences and a SEA module. Excessive shedding of transmembrane MUC12 extracellular domains is often observed for metastatic carcinoma and during inflammatory bowel disease and cystic fibrosis [21]. Zhang et al. found that high expression of MUC12 was associated with poorer overall survival (OS) in RCC and could be used as a diagnostic or prognostic marker for RCC [22]. Gao et al. described that MUC12 bore the ability to increase c‐Jun protein levels in RCC, which in turn transcriptionally regulated TGF‐β1 [23]. Above results indicated that increased MUC12 expression is a frequent event supporting growth of malignancy and inflammation. This renders the protein a promising coupling molecule for use with antibodies or other molecules targeting mRCC cells.

In this study, we found that the a lncRNA (ENSG00000225298) downregulated expression in RCC, we named this lncRNA as SLERCC (Specific Low Expression in RCC) based on next analyses. The reduced expression of SLERCC was significantly correlated with advanced tumor stage, tumor grade, and poor prognosis. Multivariate Cox regression analysis showed that SLERCC was an independent prognostic factor for OS and disease-free survival (DFS) of RCC patients. Several studies have investigated the mechanism of action of lncRNAs, including their anti-cancer roles. However, only a few lncRNAs are identified that exert tumor suppressor functionality and they are difficult to synthesize owing to their length and poor stability.

**Fig. 1:**
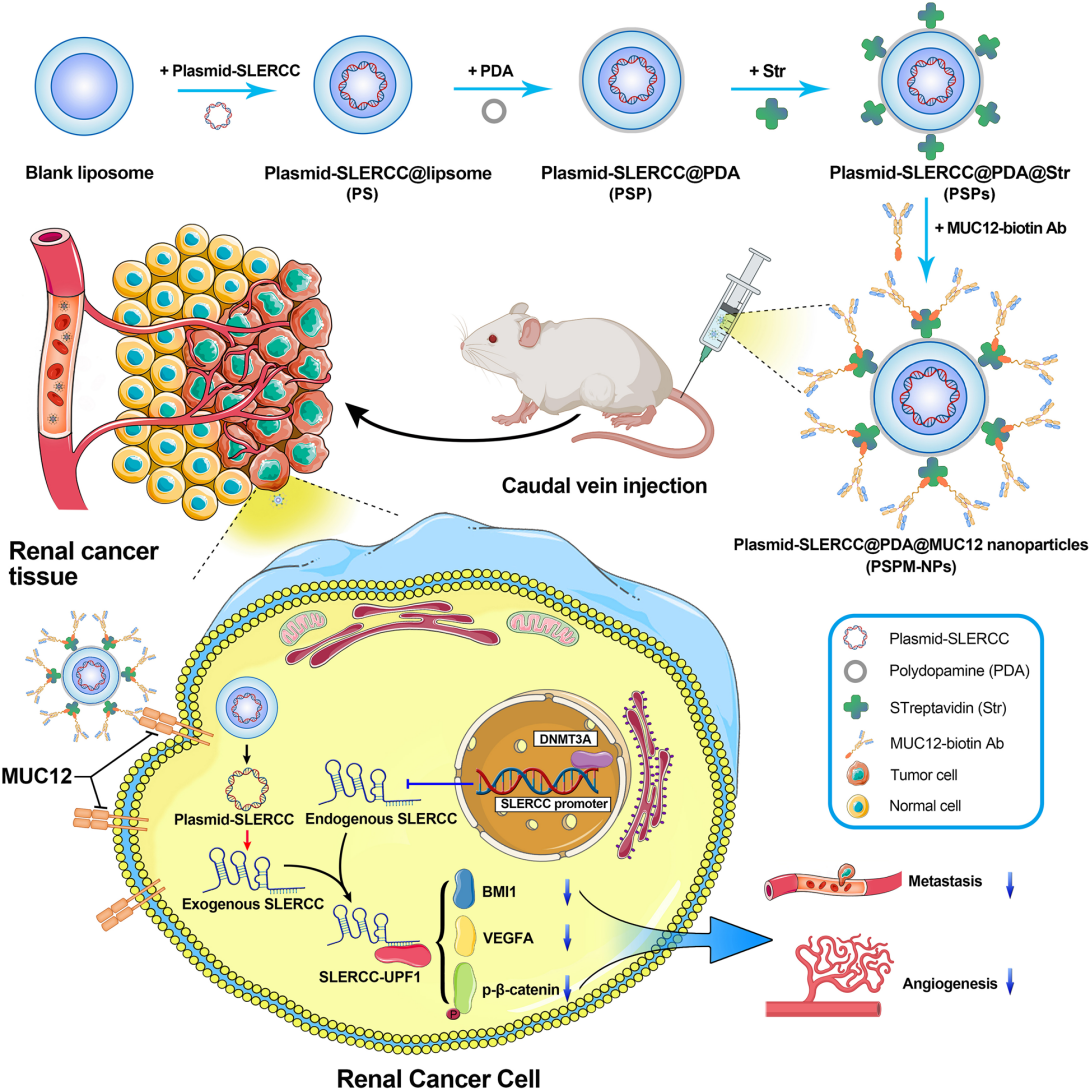
Schematic illustration of PSPM-NPs and SLERCC in RCC progression. High DNMT3A was recruited at the SLERCC promoter, which induced aberrant hypermethylation, leading to downregulation of SLERCC expression in RCC. PSPM-NPs transfer SLERCC plasmids into cells by recognizing MUC12 molecules on the RCC cell membrane surface. Elevated SLERCC can directly interact with UPF1 to activate the Wnt/β-catenin signaling pathway, which in turn inhibits RCC metastasis and angiogenesis. Abbreviations: PSPM-NPs, Plasmid-SLERCC@PDA@MUC12 nanoparticles; RCC, Renal cell carcinoma

Polydopamine (PDA) are the most widely applied polymers in chemical modification; they possessed the near-sphere structures and are immensely used in clinical transformation [24]. The surface of PDA can be modified by different kinds of reagents through the chemical bond grafting for various functions, including increasing biocompatibility, improving targeting capability and diminishing toxicity [25, 26]. In this study, we constructed the plasmid-encoding lncRNA-SLERCC to overcome the lncRNA instability, and prepared a novel Plasmid-SLERCC@PDA@MUC12 nanoparticles (PSPM-NPs) to targeted deliver SLERCC to the RCC cell lines by the incorporation of MUC12 anti-body into PDA (Fig. 1). The RCC cell lines and xenograft mouse models were conducted to evaluate the targeting delivery ability and anti-tumor mechanism of PSPM-NPs.

## Materials and methods

### Clinical specimens

RCC tissues and correspondingly matched adjacent normal tissues were obtained from 90 patients who underwent nephrectomy between January 2014 to December 2019 at the Department of Urology, Shanghai Tenth People's Hospital of Tongji University (Shanghai, China), Zhongda Hospital of Southeast University (Nanjing, China) and Beijing Chao-Yang Hospital of Capital Medical University (Beijing, China). Histopathology of all the cases of RCC was confirmed by senior pathologists and staged according to the 8th edition of the American Joint Committee on Cancer (AJCC). None of the patients received radiotherapy or other treatment modalities prior to surgery. Demographics and clinicopathological information of these patients are listed in Table S1. The protocol for the collection of tissue samples was evaluated and approved by the Ethics Committee of the Shanghai Tenth People's Hospital (SHSY-IEC-BG/02.04/04.0–81,602,469). All patients provided written informed consent.

### Cell lines and cell culture

Human RCC cell lines, 786-O (RRID: CVCL_1051), A498 (RRID: CVCL_1056), OSRC-2 (RRID: CVCL_1626), 769-P (RRID: CVCL_1050), Caki-1 (RRID: CVCL_0234), Caki-2 (RRID: CVCL_0235), ACHN (RRID: CVCL_1067), normal renal tubular epithelial cells (HK-2) (RRID: CVCL_0302), and human umbilical vein endothelial cells (HUVECs) (RRID: CVCL_2959) were purchased from the Cell Bank of the Chinese Academy of Sciences (Shanghai, China). The culture medium used for maintaining each cell line and the culture conditions were followed as described previously [3, 27]. The RCC cell lines were stored at -80 °C using CELLSAVING reagent (NCM, Suzhou, China).

### Tube formation assay

Fifty μL Matrigel (BD Biosciences, USA) was added to each well of a 96-well plate (Corning, USA) separately and allowed to incubate at 37 °C for 1 h. Next, 5000 HUVECs were seeded per well and incubated for 6 h in media containing the supernatant of the pre-treated cells. Photographs were captured and cells were counted under a microscope (Leica Microsystems, Mannheim, Germany).

### Sphere formation assay

Transfected cells were seeded into ultra-low attachment 96-well plates (Corning, USA) and cultured in DMEM-F12 media (Gibco, USA) supplemented with serum-free media containing 5 μg/mL insulin (Sigma, USA), 20 ng/mL fibroblast growth factor (Sigma, USA), and 20 ng/mL epidermal growth factor (Sigma, USA). The spheres thus formed were imaged after two weeks.

### *Fluorescence *in situ* hybridization (FISH)*

In situ hybridization assay was performed using specific probes for SLERCC and UPF1 to observe the localization of SLERCC and UPF1 in RCC cells. Briefly, 786-O and ACHN cells were grown on crawl sheets according to the manufacturer's instructions (C10910, RiboBio, Guangzhou, China). Following fixation and permeabilization, the cells were probed with cy3-labeled UPF1 and fluorescein isothiocyanate (FITC)-labeled SLERCC probes overnight at 37 °C; the nuclei were stained with DAPI.

### 5′-azacytidine (5-AZA) treatment

ACHN and 786-O cells were seeded into 12-well plates (Corning, USA) and allowed to adhere for 16 h. The cells were then treated with 5 μmol/L 5-AZA for three days, following which the media was discarded. RNA and DNA were extracted as described previously [14].

### Bisulfite sequencing PCR (BSP)

BSP was performed according to the protocol specified in the DNA methylation kit (Qiagen, cat# 59,824). Assessment of the methylation status of the SLERCC promoter region by BSP was performed by IBSBIO (Shanghai, China). The methylation patterns were evaluated using the BiQ Analyzer software.

### RNA immunoprecipitation (RIP) assay

RIP experiments were performed using the Magna RIP RNA-Binding Protein Immunoprecipitation Kit (Millipore, MA, USA) following the manufacturer's protocol. Briefly, the correspondingly treated cells were harvested and lysed using RIP lysis buffer. Samples were immunoprecipitated using magnetic beads conjugated with anti-UPF1 antibody (Abcam, MA, USA) or rabbit anti-IgG as the negative control. The purified total RNA was subsequently analyzed by qRT-PCR.

### Analysis of the TCGA-RCC dataset

The information on TCGA-RCC patients was extracted from The Cancer Genome Atlas (TCGA) database, which included data on gender, age, TNM stage, histological grade, pathological stage, survival status, survival time, lncRNAs, and transcriptome profile (Fragments Per Kilobase Million [FPKM] value). Kaplan–Meier survival curves, univariate and multifactorial Cox regression analyses were used to assess the effects of SLERCC, MUC12, and DNMT3A on OS and DFS.

### Bioinformatics

We used the online software starbase (https://starbase.sysu.edu.cn/) to predict the proteins that could potentially bind directly to SLERCC. Next, we used the catRAPID (http://service.tartaglialab.com/page/catrapid_group) website to evaluate the Z-score, interaction strength, and RNA binding domain instances for each protein, and RPISeq (http://pridb.gdcb.iastate.edu/RPISeq/) to assess the random forest (RF) and support vector machine (SVM) classifiers.

### Cell transfection

si-DNMT3A, a negative control (si-NC), and three small interfering RNA oligos (si-SLERCC#1, si-SLERCC#2, and si-SLERCC#3) specifically targeting SLERCC, were purchased from IBSBIO (Shanghai, China). The full-length cDNA of human SLERCC was synthesized by Invitrogen and cloned into the pCDNA3.1 expression mini vector. Control plasmids and plasmid-mediated SLERCC overexpression, as well as knockdown vector constructs (sh-SLERCC) and plasmid-mediated DNMT3A overexpression, were obtained from IBSBIO (Shanghai, China). For functional in vitro assays in RCC cells, transient transfection was performed using lipofectamine-3000 (Thermo Fisher Scientific, USA) at cell confluency of 30-50%. For lentiviral transduction, packaging plasmids along with sh-NC, SLERCC, or sh-SLERCC vectors were co-transfected into HEK-293 T cells and incubated for 48 h. Virus-containing supernatants were collected and added to the target cells, and finally, the infected cells were screened by puromycin selection (Gibco, USA).

### RNA sequencing

To identify RCC-associated lncRNAs, we analyzed three pairs of human RCC tissues and matched paracancerous normal tissue gene arrays. RNA sequencing was performed following the procedure described previously [3]. The screening criteria for the differential genes expression were absolute fold change ≥ 1 and false discovery rate (FDR) < 0.05.To elucidate the molecular mechanism underlying SLERCC involvement in RCC progression, we also performed an RNA sequencing of the ACHN and Caki-1 cells transfected with sh-SLERCC and sh-NC lentiviral constructs.

### RNA extraction and quantitative real-time polymerase chain reaction (qRT-PCR)

Total RNA was extracted from cells or tissue specimens stored in liquid nitrogen using the Trizol reagent (TaKaRa, China). RNA was isolated from the nuclear and cytoplasmic fractions using the NE-PER Nuclear and Cytoplasmic Extraction Reagent (Thermo Fisher Scientific, Waltham, USA). cDNA was obtained by reverse transcription using a cDNA kit (R312, Vazyme Biotech, Nanjing, China), following which qRT-PCR was performed using the SYBR Green PCR kit (Q141, Vazyme Biotech, Nanjing, China). Subsequently, the CT values of the samples were determined on the ABI Prism 7500 sequence detection system (Applied Biosystems, USA). The relative expressions of SLERCC, UPF1 and DNMT3A were calculated using the 2-ΔΔCt method, and the expression of GAPDH was used as the internal reference. All primer sequences are listed in Table S2.

### Cell Counting Kit-8 (CCK-8) assay

Pre-treated or transfected cells were inoculated in 96-well plates (Corning, USA) at a density of 2000 cells per well. After seeding cells and incubating them for 12 h, 24 h, 48 h, 72 h, and 96 h, the media were discarded and 10 µl CCK8 solution (Yeasen, Shanghai, China) in 100 µl serum-free medium was added to each well. The cells were incubated in the dark for 2 h at 37 °C, and the optical density (OD) values were measured at 450 nm using a microplate spectrophotometer (BioTek, Winooski, USA).

### 5-Ethynyl-2′-deoxyuridine (EdU) assay

Pre-treated or transfected cells were seeded in 6-well plates (Corning, USA) and cultured overnight. After incubation with 10 μM EdU reagent for 2 h, the cells were fixed with paraformaldehyde (4%), permeated with 0.5% Triton X-100 in PBS buffer, followed by washing with PBS buffers. Next, the cells were incubated with AlexaFluor-488 in the dark; nuclei were counterstained with 4,6-diamidino-2-phenylindole (DAPI), and finally, images were captured using an Olympus microscope (Tokyo, Japan).

### Wound healing assay

Transfected cells were seeded into 6-well plates (Corning, USA). When the cells attained 80% confluency, they were scratched using a 200 μL pipette tip. The debris was subsequently washed out using PBS buffer, and media supplemented with 2% fetal bovine serum was added to each well. Photographs were taken at 0 h and 24 h of wounding using an Olympus microscope (Tokyo, Japan).

### Transwell assay

Assays to assess cellular migration and invasion were performed. The upper chamber was pre-coated with Matrigel (BD Biosciences, USA) for cellular invasion experiments. Specifically, pre-treated or transfected cells were inoculated into the upper chamber, and 600 μL of 10% media was added to the lower chamber. After 12–24 h of incubation, cells in the upper chamber were removed using cotton swabs, while those on the surface of the lower chamber were fixed using anhydrous ethanol, stained with 0.1% crystalline violet (Vicmed, China), photographed, and counted using a microscope (Leica Microsystems, Mannheim, Germany).

### Western blotting, immunohistochemistry (IHC), and RNA pull‐down assay

Western blotting and IHC were performed as described previously [28, 29], and information on the antibodies used is listed in Table S3. SLERCC biotin-labeled and NC biotin-labeled probes were added to the cell lysate products, accordingly, and the complexes were subsequently incubated with 50 μl of streptavidin magnetic beads (Thermo Fisher Scientific, Inc.) at room temperature. The products were subjected to RNA extraction and purification protocols, and finally, the PCR products were analyzed by agarose gel electrophoresis and Western blotting.

### Synthesis of Plasmid-SLERCC@PDA@MUC12 nanoparticles (PSPM-NPs)

The Plasmid-SLERCC@PDA@MUC12 nanoparticles (PSPM-NPs) were synthesized by a rapid and green method. Briefly, 50 nM SLERCC plasmid dissolved in RNase free water was loaded into the commercial liposomes (50 μL; Yeasen, China) by vortexing for 30 s to form Plasmid-SLERCC@lipsome (PS). The resulting suspension was dispersed in 5 mL Tris–HCl (pH 8.8; 10 mM) solution with the subsequent addition of dopamine hydrochloride (5 mg) resulting in the formation of polydopamine (PDA) (Adamas-beta Inc. China) modified liposomes after stirring for 3 h. Next, the PDA-modified liposomes (Plasmid-SLERCC@PDA, PSP) were collected by centrifugation at 8,000 rpm for 10 min and washed using distilled water. Next, the obtained mixtures were dispersed in 1 ml streptavidin solution (2 mg/mL; dissolved in PBS with the pH 8 ~ 9) and shaken thoroughly for 24 h in dark at 4 ℃ resulting in the synthesis of Plasmid-SLERCC@PDA@Str (PSPs). For PSPM-NPs synthesis, the obtained PSPs was mixed with 1 ml biotinylated MUC12 antibody solution (50 μg/mL; Bioss, China) and incubated for 1 h. The obtained suspension was washed using PBS buffer and stored at 4 ℃ till further use.

### Characterization of NPs

Imaging by transmission electron microscopy (TEM) and scanning transmission electron microscopy (STEM) with energy dispersive X-ray spectrometry (EDS) element mapping was performed to characterize PSPM-NPs. The sample was placed on the carbon cover copper TEM grids and photographed by TEM (JEOL, Tokyo, Japan).

### Determination of particle size potential of NPs

Separately, 20 μL of PSP and PSPM-NPs were dissolved in 1 mL distilled water. The zeta potentials and sizes of the samples were determined using a particle size potentiometer (Nano ZS90, Worcestershire, UK).

### Encapsulation capability of SLERCC plasmid

The encapsulation of SLERCC plasmid was examined by gel electrophoresis. Using empty plasmid as a negative control, PSP and PSPM were separately examined to determine their RNA encapsulation capability. The gels were prepared by mixing 0.6 g agarose in 30 ml 1 × TAE buffer. After mixing with 2 μL 6 × DNA loading buffer and 1 μL SYBR Green I nucleic acid gel staining solution, 9 μL samples were loaded in the dented pores. Electrophoresis voltage was set at 120 V for 20 min. Subsequently, the gel was imaged and analyzed on the Tanon Gel image system (Shanghai, China).

### Measuring MUC12 incorporation

The incorporation of MUC12 was measured by western blot analysis. After adding 4 μL 5 × protein loading buffer, 16 μL samples were heat-denatured by boiling for 30 min at 100℃. Next, PSP and PSPM-NPs samples were loaded into the 10% sodium dodecyl sulfate–polyacrylamide gel (SDS-PAGE) for electrophoresis with PDA as the negative control. After electrophoresis for 2 h, the gels were dyed using Coomassie blue staining buffer for 3 h and washed by distilled water, and stored overnight. On the next day, the gels were photographed; the MUC12 band was observed at ~ 62 kDa.

### Cytophagy of PSPM-NPs

For successful plasmid delivery, the cytophagy of PSPM-NPs was examined by bio-TEM imaging. ACHN cells were cultured in a 6-well plate for 16 h till they reached 70 ~ 80% confluency. The cells were incubated for 6 h with 40 μL SPM diluted in 2 mL DMEM media and washed using distilled water. Next, the cells were trypsinized and collected by centrifugation. The cells were fixed with glutaraldehyde fixative (2.5%) at stored overnight at 4℃. The prepared samples were washed and dehydrated before polymerization using Spurr’s low-viscosity solution at 60℃ for two days. Finally, the samples were sliced and stained with lead citrate before bio-TEM imaging.

### Animal model

A total of 60 male BALB/c-nu mice, aged 4–6 weeks, were purchased from Slac Laboratory Animal (Shanghai, China). All mice were housed in a pathogen-free environment, and all the animal experiments were performed in accordance with the protocol approved by the Animal Research Ethics Committee of the Shanghai Tenth People's Hospital. The length and width of the tumors in mice were measured weekly, and the tumor volume was calculated using the following formula: volume (mm3) = 0.5 × width2 × length. After sacrificing, the weights of each tumor from all mice were recorded.

### Subcutaneous xenograft model

a) 100 μl of 5 × 10^7^ ACHN cells were mixed with 100 μl of Matrigel (BD, USA) and injected subcutaneously into mice (4 groups, *n* = 3 in each group). 3 weeks later, PBS, Sunitinib, PSPM-NPs or PSPM-NPs + Sunitinib (both 10 nmol) were injected intravenously three times a week (200 μl) for two weeks. b) 100 μl of 5 × 10^7^ sh-NC and sh-SLERCC#3 stably transfected ACHN cell lines were mixed with 100 μl of Matrigel (BD, USA) and injected subcutaneously into mice (2 groups, *n* = 4 in each group). Tumor size and tumor changes were observed.

### Tail vein lung metastasis model

Two hundred μl of 1 × 10^6^ ACHN cells were injected into the tail vein of each mouse (4 groups, *n* = 6 in each group). 3 weeks later, PBS, Sunitinib, PSPM-NPs, or PSPM-NPs + Sunitinib (both 10 nmol) were injected intravenously three times a week (200 μl) for two weeks, respectively. Tumor progression was observed on an IVIS imaging system (Calipers, Hopkinton, USA).

### Orthotopic xenograft model

1 × 10^6^ sh-NC and sh-SLERCC#3 stably transfected ACHN cell lines were orthotopically implanted into mice (2 groups, *n* = 5 in each group) in the subrenal positions of both kidneys. Tumor progression was observed on an IVIS imaging system (Calipers, Hopkinton, USA).

### In vivo biocompatibility of PSPM-NPs

Six male BALB/c mice were randomly divided into two groups (2 groups, *n* = 3 in each group). The control group and PSPM-NPs group were injected with 200 μl of PBS or PSPM-NPs (both 10 nmol), respectively. Mice were sacrificed at 15 days, and lung, liver, spleen, kidney, heart and colon were harvested for subsequent HE staining and IHC staining.

### Statistical analysis

R-Studio (Boston, USA), SPSS 20.0 (RRID:SCR_002865, Inc., Chicago, USA), and GraphPad Prism 8.3 software (San Diego, USA) was used for statistical analyses. Two-tailed Student's t-test or χ2 test was used to assess differences between components. Survival analysis was performed using the Kaplan–Meier method and significance was confirmed by a log-rank test. Univariate and multivariate Cox proportional hazards models were used for survival analyses. *P*-values < 0.05 were considered statistically significant.

## Results

### SLERCC is downregulated in RCC tissues and associated with a favorable prognosis

**Fig. 2:**
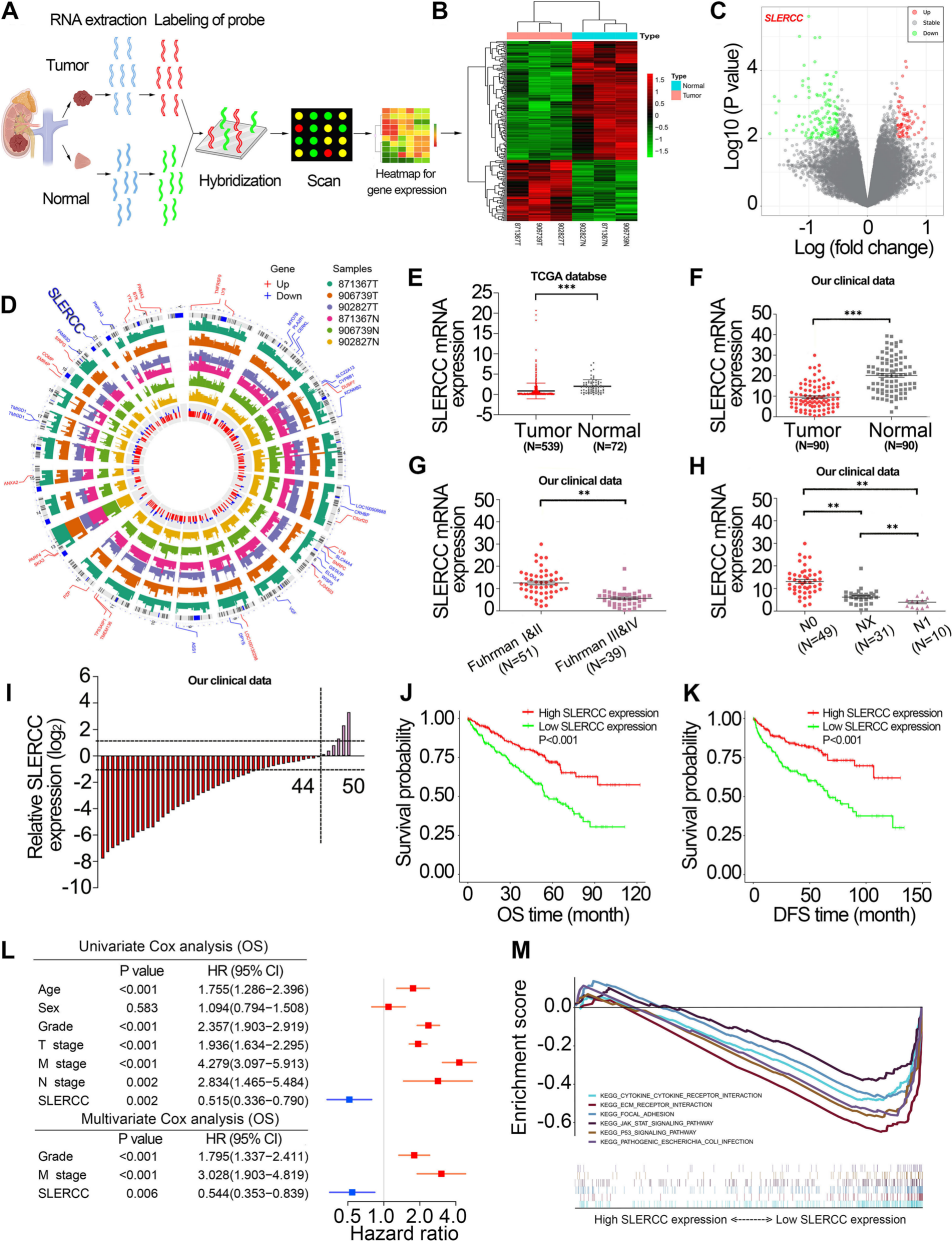
SLERCC is downregulated in RCC tissues. **A** The process of gene microarray. **B**, **C** The heatmap and volcano plot of differentially expressed genes. **D** The expression levels and chromosome positions of the differentially expressed genes. **E** Expression of SLERCC in normal (*n* = 72) and tumor (*n* = 539) tissues in TCGA dataset. **F** Relative expression of SLERCC in 90 paired tissue samples in our clinical data. **G**, **H** Relative expression levels of SLERCC in tissue samples for tumor Fuhrman grade (**G**) and N stage (**H**) in our clinical data. **I** Relative expression of SLERCC in 50 paired tissue samples in our clinical data. **J**, **K** Overall survival (**J**) and disease-free survival (**K**) curves for RCC patients with low and high SLERCC expression in TCGA dataset. **L** Univariate and multivariate Cox regression of OS for SLERCC and other clinicopathological features in TCGA dataset. **M** GSEA analysis of different SLERCC expression levels. (**p < 0.01, ***p < 0.001). Abbreviations: OS, Overall survival; DFS, Disease-free survival; TCGA, The Cancer Genome Atlas; RCC, Renal cell carcinoma

To identify the important lncRNAs underlying RCC progression, we analyzed three pairs of human RCC tissues and matched paracancer normal tissue gene arrays (Fig. 2A). A total of 198 differentially expressed genes were identified, including 67 upregulated and 131 downregulated genes (Fig. 2B), and the chromosomal locations of all differentially expressed genes are displayed in Fig. 2D. SLERCC was the most significantly downregulated lncRNA (Fig. 2C). Next, we examined the expression of SLERCC in different tumor types and found its levels were downregulated in KIRP, RCC and KIRP specimens in the TCGA database (Fig. S1A). Subsequently, we analyzed the expression of SLERCC in 539 RCC tissues and 72 normal tissuess in the TCGA database and found that the expression of SLERCC was downregulated in RCC tissues (Fig. 2E and Fig. S1B). SLERCC expression was further reduced in stage III/IV (Fig. S1C), and receiver operating characteristic (ROC) curve showed that SLERCC expression had high accuracy in distinguishing RCC tissues from its normal counterpart (area under the curve (AUC) = 0.799, Fig. S1D). In addition, the chi-square test results suggested that the expression of SLERCC was correlated with T stage, M stage, histological grade, and pathological stage in TCGA dataset (Table S4). Furthermore, qRT-PCR analysis of SLERCC expression in 90 pairs of RCC and correspondingly matched normal tissues in our clinical data showed that SLERCC expression was significantly reduced in tumor tissues (Fig. 2F, I), and was substantially negatively correlated with Fuhrman grade and N stage (Fig. 2G, H).

Kaplan–Meier survival curves suggested that RCC patients with low SLERCC expression had significantly lower OS and DFS relative to those with high SLERCC expression (Fig. 2J, K). In addition, multivariate Cox survival analysis showed that SLERCC expression was an independent risk factor for OS (HR = 0.544, 0.353–0.839 [95% CI], *P* = 0.006) (Fig. 2L and Table S5) and DFS (HR = 0.526, 0.309–0.893 [95% CI], *P* = 0.017) (Fig. S1E and Table S6) of RCC patients. Moreover, we constructed a nomogram of OS prognosis for RCC patients including SLERCC expression status based on the results of multivariate Cox survival analysis (Fig. S1F). GSEA analysis showed that ECM receptor interaction, focal adhesion, JAK/STAT signaling pathway and P53 signaling pathway were mainly enriched in the low SLERCC expression group (Fig. 2M). Together, these data supported the expression of SLERCC as a protective factor for predicting the survival of RCC patients.

### Syntheses and characteristics of PSPM-NPs

MUC12 is a transmembrane glycoprotein localized mainly on the cell membrane. We examined the expression of MUC12 in different tumor types and found that its expression was upregulated in RCC in the TCGA database (Fig. S2A) and that high expression of MUC12 was an independent risk factor for OS and DFS in RCC patients (Fig. S2B, C). Kaplan–Meier survival curves indicated that high expression of MUC12 was associated with poor OS and DFS (Fig. S2D, E). Subsequently, we analyzed the expression of MUC12 in the TCGA database and found that MUC12 was highly expressed in tumor tissues and that MUC12 expression correlated with T-stage, N-stage, M-stage, histological grade and pathological stage (Fig. S2F-K). In addition, IHC staining showed that MUC12 expression was increased in tumor as well as metastatic samples, and MUC12 expression increased with increasing tumor stage (Fig. S3).

**Fig. 3:**
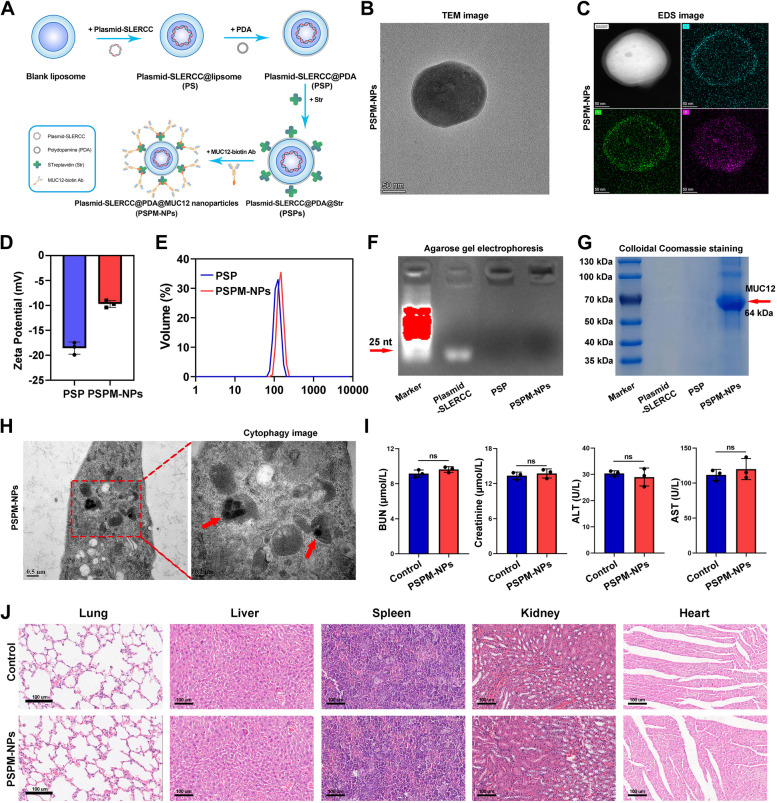
Preparation and characterization of PSPM-NPs. **A** Strategies for preparing MUC12-targeted PSPM-NPs. **B** TEM mapping of PSPM-NPs. **C** EDS imaging for PSPM-NPs. **D** Zeta potentials of PSP and PSPM-NPs. **E** Elevated diameters of PSP and PSPM-NPs. **F**, **G** Agarose gel electrophoresis (**F**) and colloidal Coomassie images (**G**) of PSP and PSPM-NPs. **H** Images of PSPM-NPs-mediated cytophagocytosis of ACHN cells. **I** (c) Serum analysis of BUN, creatinine, ALT, and AST in mice after PSPM-NPs i.v. injection. **J** H&E staining of lung, liver, spleen, kidney and heart in mice treated with PSPM-NPs. Abbreviations: PSPM-NPs, Plasmid-SLERCC@PDA@MUC12 nanoparticles; PSP, Plasmid-SLERCC@PDA; EDS, Energy dispersive X-ray spectrometry; TEM, Transmission electron microscopy; BUN, Blood urea nitrogen; ALT, Alanine aminotransferase; AST, Aspartate aminotransferase

The strategy for preparing MUC12-targeted PSPM-NPs is schematically illustrated in Fig. 3A. TEM imaging and EDS mapping exhibited that the synthetic PSPM-NPs had a near-spherical shape upon successful modification by PDA (Fig. 3B, C). The decreased zeta potentials and elevated diameter suggested that MUC12 was grafted in the surface of PSP and that the PSPM-NPs had good stability with an average diameter of approximately 140 nm (Fig. 3D, E). Agarose gel electrophoresis and colloidal Coomassie staining results showed that SLERCC plasmid was successfully wrapped and the MUC12 modification was present on the surface of PSPM-NPs (Fig. 3F, G). The cytophagocytosis experiments showed that PSPM-NPs could be successfully internalized into the ACHN cells for successful plasmid delivery (Fig. 3H). We then examined the in vivo biocompatibility of PSPM-NPs in normal mice. The results showed that blood urea nitrogen (BUN), creatinine, alanine aminotransferase (ALT), and aspartate aminotransferase (AST) were within the normal range after PSPM-NPs injection compared to controls (Fig. 3I), and no significant morphological changes occurred in HE staining of major organs such as lung, liver, spleen, kidney and heart (Fig. 3J). These results suggest that PSPM-NPs have little systemic toxicity.

### *SLERCC as a tumor suppressor inhibits the progression of RCC cells *in vitro

**Fig. 4:**
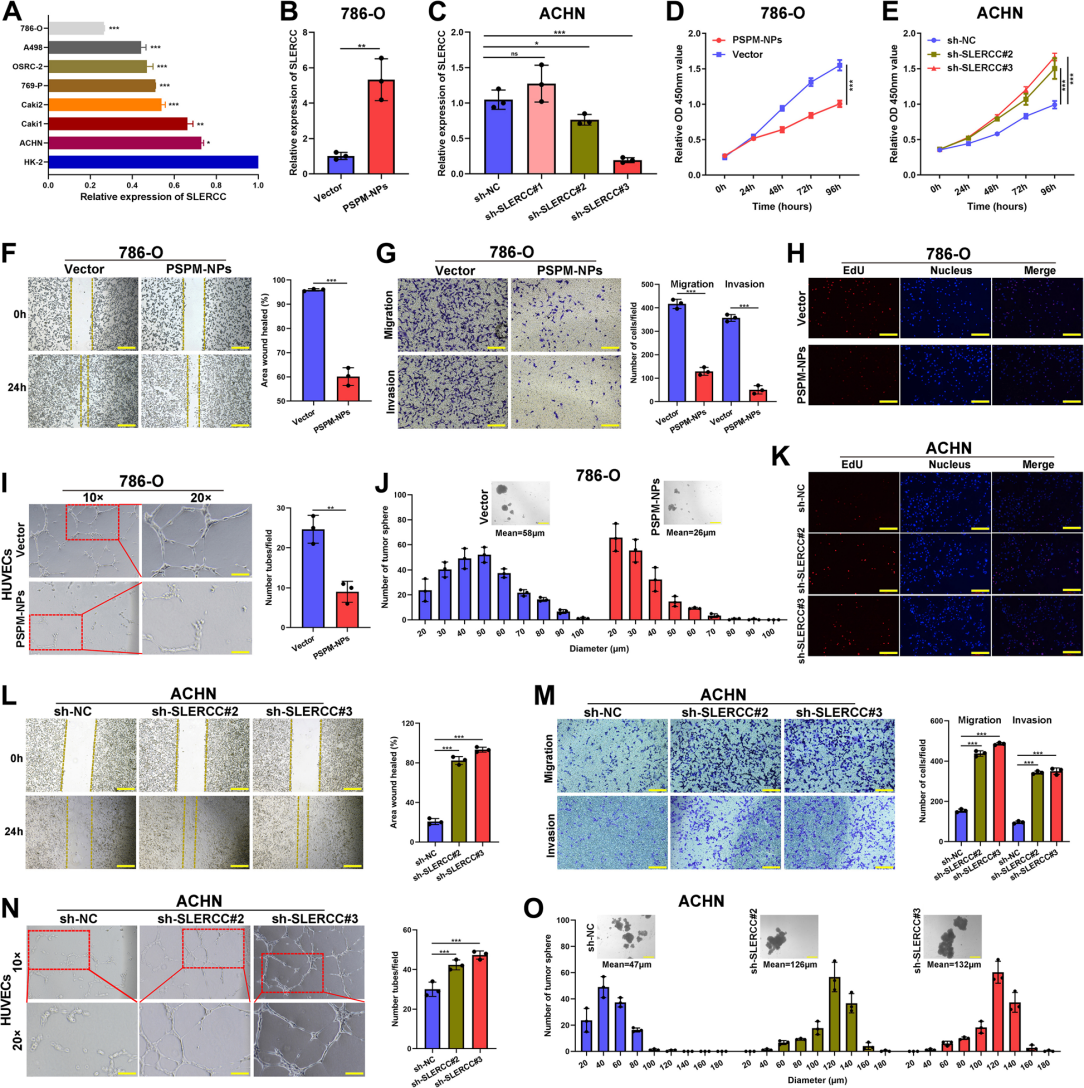
SLERCC as a tumor suppressor inhibits the progression of RCC cells in vitro. **A** Relative expression of SLERCC in different ccRCC cell lines. **B** Relative expression of SLERCC in 786-O cell lines transfected with PSPM-NPs or Vector. **C** Relative expression of SLERCC in ACHN cell lines transfected with sh-NC, sh-SLERCC#1, sh-SLERCC#2, or sh-SLERCC#3 constructs. **D**-**E** Growth curves of 786-O cells (**D**) and ACHN cells (**E**) were measured after transfection with indicated vectors by CCK-8 assays. **F**, **L** The migratory capacity of 786-O cells (**F**) and ACHN cells (**L**) transfected with indicated vectors assessed by the wound healing assay. **G**, **M** Cell migration and invasion of 786-O cells (**G**) and ACHN cells (**M**) transfected with indicated vectors assessed by Transwell migration and Matrigel invasion assays. **H**, **K** Growth curves of 786-O cells (**H**) and ACHN cells (**K**) transfected with indicated vectors assessed by EdU assays. (**I**, **N**) The angiogenic capacity of 786-O cells (**I**) and ACHN cells (**N**) transfected with indicated vectors assessed by tube formation assay. **J**, **O** Stemness of 786-O cells (**J**) and ACHN cells (**O**) transfected with indicated vectors assessed by the tumorsphere assay. (*p < 0.05, **p < 0.01, ***p < 0.001)

To elucidate the biological roles of SLERCC in RCC progression, first, the expression of SLERCC in different RCC cell lines was assessed. SLERCC was expressed at low levels in RCC cell lines (Fig. 4A). We constructed a lentivirus overexpressing SLERCC (Fig. S4A-D) and found that PSPM-NPs had similar transfection ability to conventional overexpressing lentiviruses (Fig. S4E). Subsequently, qRT-PCR analysis confirmed a significant increase in SLERCC levels in 786-O cells after transfection with PSPM-NPs (Fig. 4B), and both ACHN and Caki-1 cells transfected with sh-SLERCC#2, and sh-SLERCC#3 had significantly reduced expression of SLERCC (Fig. 4C and Fig. S5A). The results of the CCK-8 and EdU experiments showed that forced expression of SLERCC significantly inhibited the proliferation profile of 786-O cells (Fig. 4D, H ), and reduced SLERCC expression significantly promoted the proliferation of ACHN and Caki-1 cells (Fig. 4E, K and Fig. S5B, C). Wound healing and Transwell assays showed that overexpression of SLERCC could significantly suppress the migration and invasive of 786-O cells (Fig. 4F, G), while knockdown of SLERCC enhanced the migration and invasion of ACHN and Caki-1 cells (Fig. 4L, M and Fig. S5D, F). Moreover, overexpression of SLERCC significantly reduced the size of tumorspheres and decreased the stemness of 786-O cells (Fig. 4J). To determine the effects of SLERCC on the angiogenic capacity, when human umbilical vein endothelial cells (HUVECs) were cultured in cell medium overexpressing SLERCC, the tube formation and growth were significantly impaired (Fig. 4I). However, decreasing SLERCC expression was able to increase the size of tumorspheres and enhance tube formation and growth of ACHN and Caki-1 cells (Fig. 4N, O and Fig. S5E, G). The showed that SLERCC as a tumor suppressor inhibited the progression of RCC cells.

### DNMT3A-mediated SLERCC promoter hypermethylation results in the downregulation of SLERCC expression in RCC cells

**Fig. 5:**
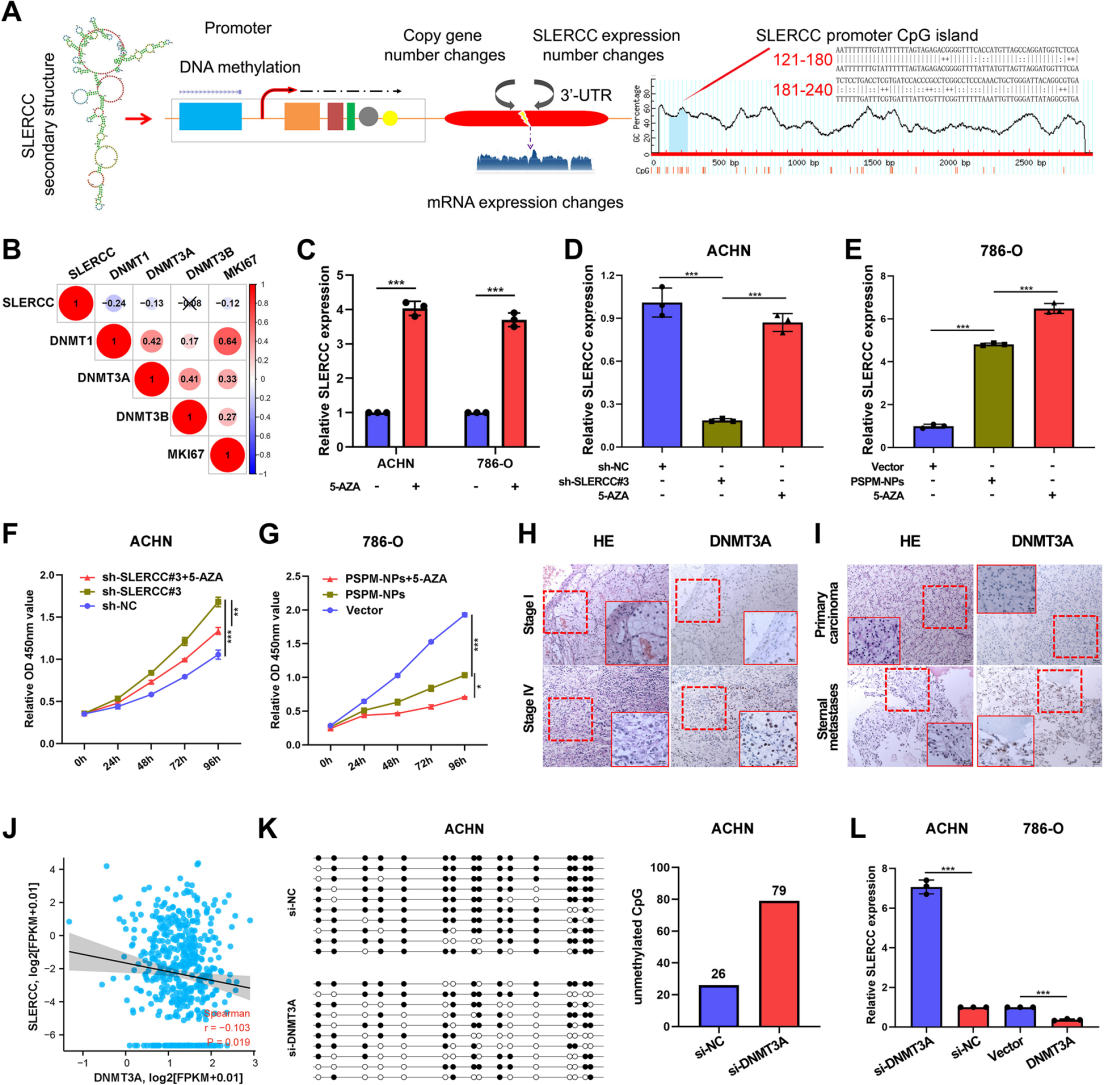
DNMT3A-mediated SLERCC promoter hypermethylation results in the downregulation of SLERCC in RCC cells. **A** CpG islands of SLERCC as predicted by MethPrimer 2.0. **B** Relationship of DNMT1, DNMT3A, and DNMT3B with SLERCC in TCGA dataset. **C**-**E** Expression of SLERCC in 786-O and ACHN cells after 5-AZA treatment. **F**, **G** Growth curves of 786-O cells (**F**) and ACHN cells (**G**) were measured after transfection with indicated vectors by CCK-8 assays. **H**, **I** IHC staining for DNMT3A in stages I and IV, primary carcinoma, and sternal metastatic tissues. **J** Relationship between DNMT3A and SLERCC in the TCGA dataset. **K** BSP assay detects DNA methylation levels of DNMT3A at CpG islands in the SLERCC promoter region in ACHN cells. **L** Relative expression of SLERCC confirmed by qRT-PCR in 786-O and ACHN cell lines transfected with si-DNMT3A, si-NC, vector or DNMT3A. (*p < 0.05, **p < 0.01, ***p < 0.001). Abbreviations: TCGA, The Cancer Genome Atlas; IHC, immunohistochemistry

Recent studies suggest that promoter CpG methylation is an early event in the process of carcinogenesis and can also promote tumorigenesis [30, 31]. SLERCC is located on chromosome 21 and MethPrimer 2.0 predicts the presence of CpG islands between 123–243 bp in the SLERCC promoter region (Fig. 5A). Given that DNA methyltransferases (DNMTs) are critical for DNA methylation, we examined the relationship of DNMT1, DNMT3A, and DNMT3B with SLERCC expression. The results suggested that the levels of DNMT1 and DNMT3A were negatively correlated with SLERCC expression (Fig. 5B, J and Fig. S6A). To confirm if the level of expression of SLERCC was regulated by the promoter methylation, first, we treated the 786-O and ACHN cells with 5′-azacytidine (5-AZA). qRT-PCR analysis showed that the level of expression of SLERCC in 786-O and ACHN cells after 5-AZA treatment was significantly upregulated (Fig. 5C). In ACHN cells, 5-AZA rescued the inhibitory effect of sh-SLERCC#3 on SLERCC, and 5-AZA enhanced the effect of PSPM-NPs on SLERCC overexpression in 786-O cells and promoted the proliferative capacity of ACHN and 786-O cells (Fig. 5D-G). Subsequently, the levels of expression of DNMT1 and DNMT3A in stages I and IV, primary carcinoma as well as metastatic carcinoma were assessed by IHC, TCGA databases and Kaplan–Meier survival curves; a significant difference was found in the expression of DNMT3A (Fig. 5H, I and Fig. S6B, F–H), while DNMT1 differences were not significant (Fig. S6C-E, I-K). Next, BSP assay demonstrated that knockdown of DNMT3A in ACHN cells significantly attenuated DNA methylation level at the CpG island of SLERCC promoter region, and vice versa in 786-O cells when DNMT3A was upregulated (Fig. 5K and Fig. S6L). In additon, we assessed whether DNMT3A could affect the level of expression of SLERCC. qRT-PCR analysis suggested that SLERCC expression was upregulated after the expression of DNMT3A was knocked down in ACHN cells, and vice versa in 786-O cells when DNMT3A was overexpressed (Fig. 5L). Furthermore, The above results suggested that the expression of SLERCC can be regulated by methylation through DNMT3A.

### SLERCC directly binds to UPF1

**Fig. 6:**
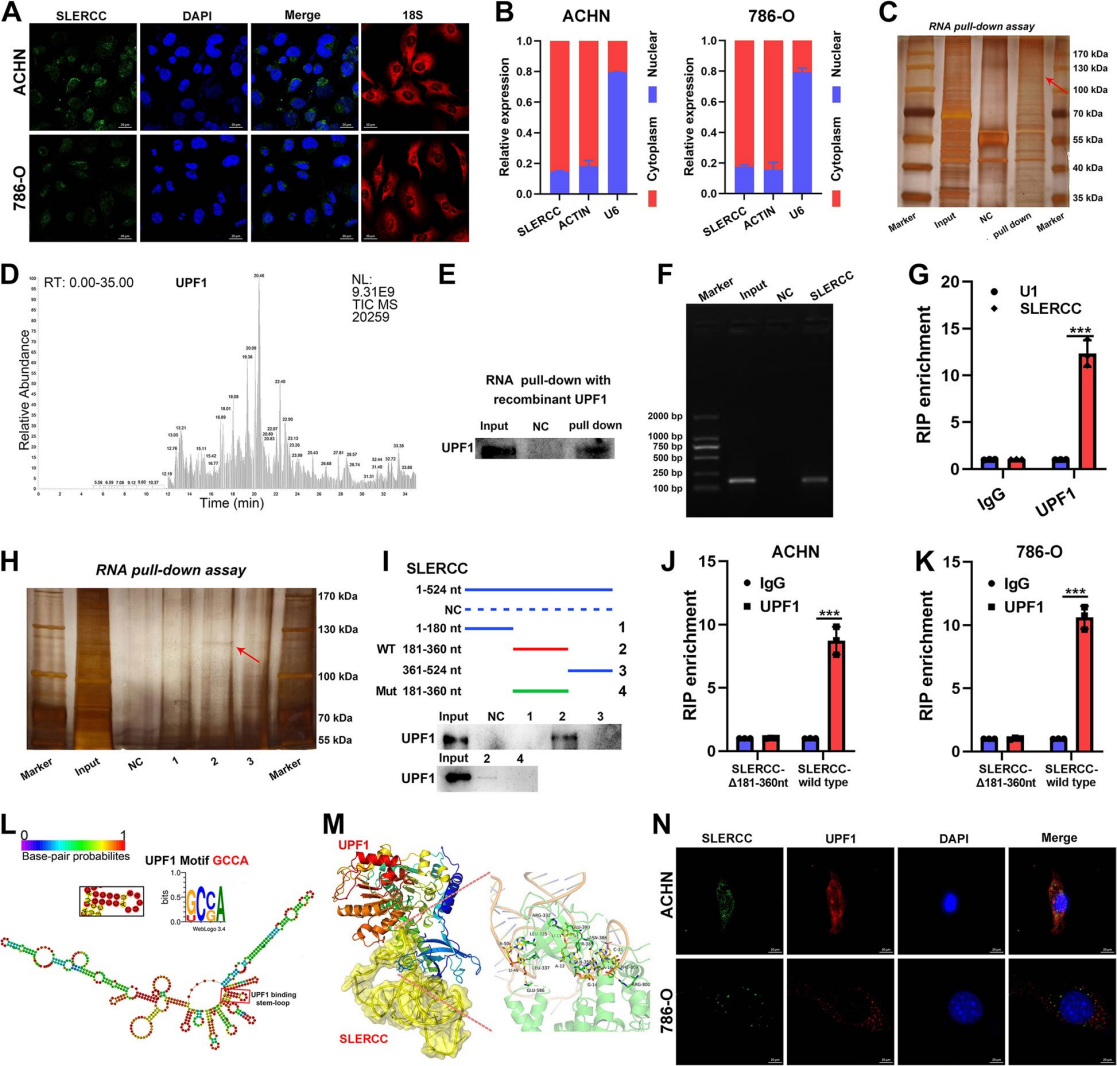
SLERCC directly binds to UPF1. **A** FISH assay to assess the intracellular localization of SLERCC in ACHN and 786-O cells. **B** qRT-PCR analysis of SLERCC in nuclear and cytoplasmic fractions of ACHN and 786-O cells. **C** RNA pull-down assay for SLERCC in ACHN cells. **D** Mass spectrometric (MS) analysis of the proteins from the RNA pull-down assay. **E** Western blotting after RNA pull-down with recombinant UPF1. **F** Agarose gel electrophoresis for the RNA pull-down assay shows that UPF1 is a direct target of SLERCC. **G** RIP assay using anti-UPF1 to assess the enrichment of SLERCC by UPF1. IgG is the negative control, while U1 is a nonspecific control. **H**, **I** Serial deletion RNA pull-down assay for SLERCC in ACHN cells. **J**, **K** RIP assay after deletion of 181–360 nt from SLERCC in ACHN and 786-O cells. **L** Prediction of the stem-loop structures at UPF1-binding sites in SLERCC. **M** PyMOL software displaying the interaction between SLERCC and UPF1 in their 3D protein structures. **N** FISH to assess the colocalization of SLERCC and UPF1 in ACHN and 786-O cells. (***p < 0.001). Abbreviations: TCGA, The Cancer Genome Atlas; FISH, Fluorescence in situ hybridization; RIP, RNA immunoprecipitation

To identify the downstream targets of SLERCC, firstly, we examined the intracellular localization of SLERCC. FISH and nuclear-cytoplasmic separation assays showed that SLERCC was expressed in both the nucleus and cytoplasmic fractions but was mainly localized to the cytoplasm (Fig. 6A, B). Based on the currently available information, lncRNAs can interact with proteins within the cell and exert their functions. We predicted eight proteins that could putatively interact with SLERCC by bioinformatics (starBase). Subsequently, we evaluated the scores of the aforementioned eight proteins using online software, catRAPID and RPISeq, and the results suggested that UPF1 had a good Z-score (5.21), interaction strength (99.18%), as well as, SVM classifier (0.98) (Fig. S7A, B). In vitro RNA pull-down assays with biotinylated SLERCC and corresponding controls led to the identification of a differential band between 100 and 130 kDa (Fig. 6C) which was verified as UPF1 by mass spectrometry (MS) (Fig. 6D). Moreover, western blot analysis and agarose gel electrophoresis after RNA pull-down assays confirmed the interaction between SLERCC and UPF1 (Fig. 6E, F). The results of the RIP assay showed that UPF1 was significantly enriched by SLERCC relative to the negative control (Fig. 6G). catRAPID suggested the presence of UPF1 binding sites in the 200-300nt long sequence of SLERCC (Fig. S7C, D). Further, the serial deletion assays confirmed that the 181-360nt long sequence of SLERCC was essential for its interaction with UPF1 (Fig. 6H, I). In addition, RIP assays showed that direct deletion of 181-360nt long region of SLERCC resulted in a significant reduction in the enrichment of UPF1 by SLERCC (Fig. 6J, K).

Next, sequence analysis by POSTAR2 suggested the presence of a sequence motif and structural preference for the RBP binding site for UPF1, located in the 230-270nt region of SLERCC in the form of a stem-loop structure (Fig. 6L). PyMOL software was used to determine the 3D structures of SLERCC and UPF1 (Fig. 6M). Subsequently, fluorescence staining confirmed the co-localization of SLERCC and UPF1 in both 786-O and ACHN cells (Fig. 6N). Additionally, we analyzed the expression of UPF1 in TCGA datasets and found that UPF1 expression was negatively correlated with SLERCCexpression (Fig. S7F), and UPF1 was downregulated in the RCC tissue and correlated with staging (Fig. S7G, H). IHC also confirmed that UPF1 expression was downregulated and negatively correlated with tumor stage (Fig. S7E).

### *SLERCC overexpression inhibits RCC progression and metastasis *in vivo

**Fig. 7:**
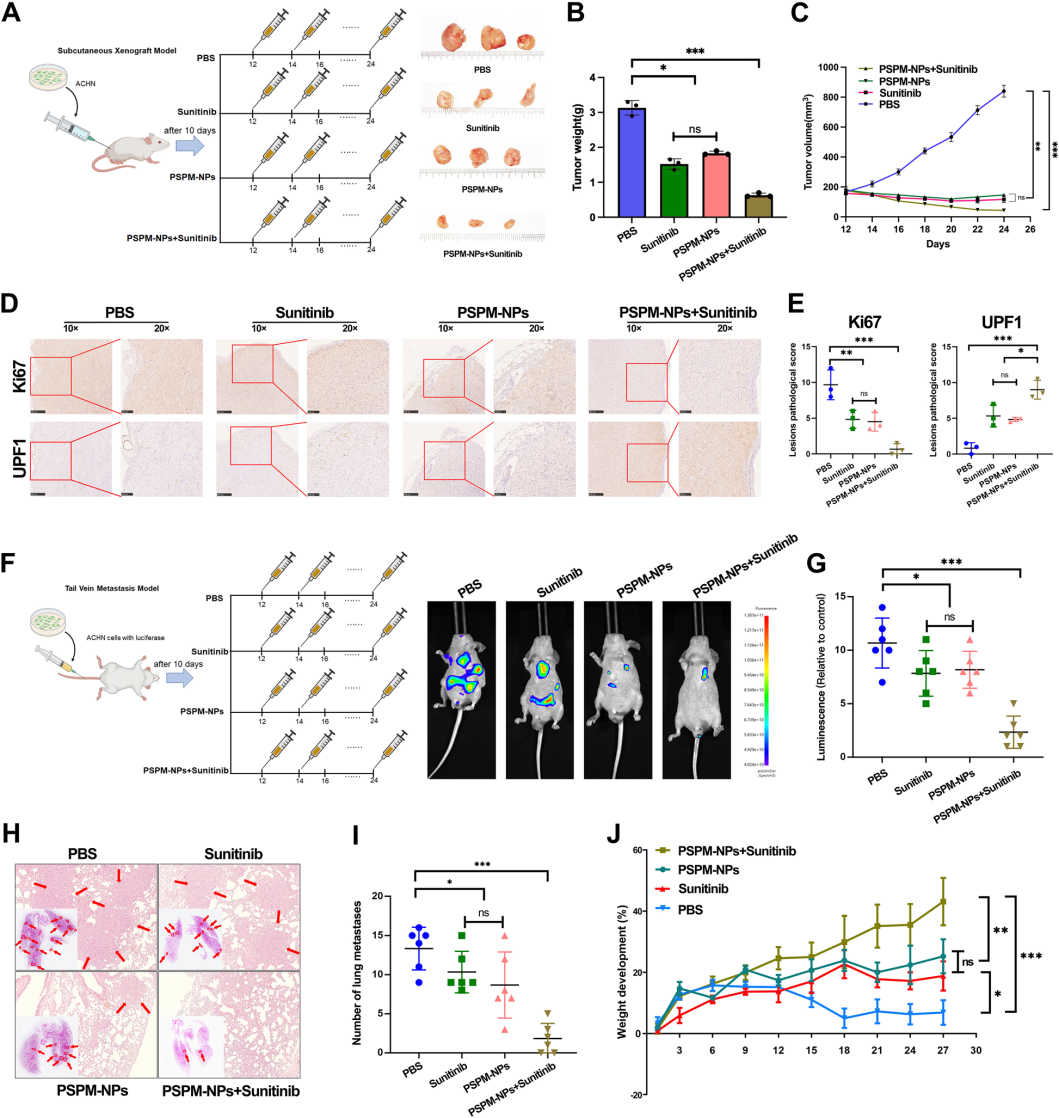
PSPM-NPs inhibit the progression and metastasis of RCC in vivo. **A** Representative bioluminescence images of subcutaneous xenograft model with intravenous injection of PBS, Sunitinib, PSPM-NPs and PSPM-NPs + Sunitinib. **B**, **C** Tumor weight (**B**) and volume (**C**) in the subcutaneous xenograft model. **D** Representative IHC images of subcutaneous xenograft model. **E** IHC scores of Ki67 and UPF1 in the subcutaneous xenograft model. **F** Representative bioluminescence images of tail vein metastasis model with intravenous injection of PBS, Sunitinib, PSPM-NPs and PSPM-NPs + Sunitinib. **G**-**I** Lumineacence (**G**) and lung matastases (**H**, **I**) in the tail vein metastasis model. **J** Tumor weight in the tail vein metastasis model. (*p < 0.05, **p < 0.01, ***p < 0.001). Abbreviations: PSPM-NPs, Plasmid-SLERCC@PDA@MUC12 nanoparticles; RCC, Renal cell carcinoma

To investigate the role of SLERCC in vivo, we first evaluated the effect of PSPM-NPs on tumors (Fig. 7A). The subcutaneous xenograft model showed that both Sunitinib and PSPM-NPs were able to suppress tumor weight and volume compared to the PBS group, and the combination of PSPM-NPs and Sunitinib significantly reduced tumor weight and volume (Fig. 7B, C). IHC results showed that the PBS group had the highest Ki67 score, while the PSPM-NPs + Sunitinib group had the highest UPF1 scores (Fig. 7D, E). Tail vein metastasis model showed that the combination of PSPM-NPs and Sunitinib significantly suppressed the luminescence intensity and number of nodules in lung metastatic tumors, with the Sunitinib and PSPM-NPs groups follow and the PBS group being the worst (Fig. 7F-I). In addition, the growth curves showed that treatment with PSPM-NPs or Sunitinib increased the weight of mice compared with the control group, and the combination of PSPM-NPs and Sunitinib prominently increased the weight (Fig. 7J).

In addition, we evaluated the effect of SLERCC knockdown on tumors in vivo. IVIS assays in an orthotopic xenograft model showed that tumors in mice injected with sh-SLERCC#3 cells had stronger luminescence intensity, higher tumor weights, higher Ki67 scores and lower UPF1 scores relative to mice injected with sh-NC cells (Fig. S8A-E). Subcutaneous xenograft model also showed that knockdown of SLERCC significantly promoted tumor volume, weight and Ki67 expression in xenografts as well as suppressed UPF1 expression (Fig. S8F-J).

### SLERCC directly interacts with UPF1 to inhibit RCC progression through the Wnt/β-catenin signaling pathway

**Fig. 8:**
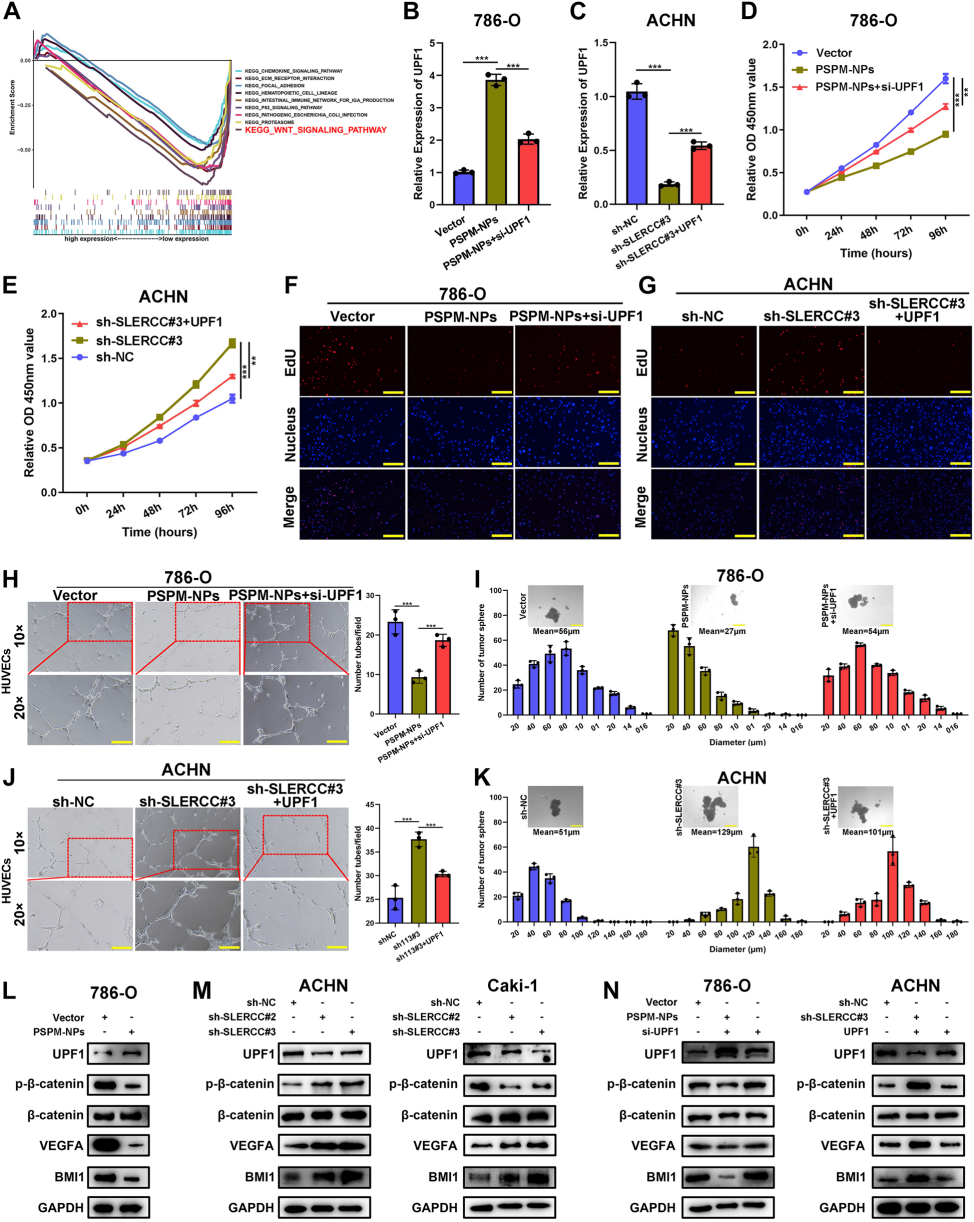
SLERCC directly interacts with UPF1 to inhibit RCC progression through the Wnt/β-catenin signaling pathway. **A** KEGG enrichment analysis for differentially expressed genes. **B**, **C** Relative expression of UPF1 as confirmed by qRT-PCR in 786-O and ACHN cell lines. **D**, **G** The rescue effects of UPF1 on the cell proliferation in SLERCC expressing 786-O and ACHN cells as assessed by CCK8 and EdU assays. **H**, **J** The rescue effect of UPF1 on the angiogenic capacity of SLERCC expressing 786-O and ACHN cells as assessed by the tube formation assay. **I**, **K** The rescue effect of UPF1 on the stemness of SLERCC in 786-O and ACHN cells as assessed by tumorsphere assay. **L** Western blotting to detect the changes in the expression of individual proteins after overexpressing SLERCC in 786-O cells. **M** Western blotting to detect changes in the expression of individual proteins after knocking down SLERCC in ACHN and Caki-1 cells. **N** Western blotting to detect the rescue effects of UPF1 on SLERCC-mediated changes in the expression of individual proteins in 786-O and ACHN cells. (**p < 0.01, ***p < 0.001). Abbreviations: RCC, Renal cell carcinoma

To explore the molecular mechanism underlying SLERCC involvement in RCC progression, we constructed sh-SLERCC#3, sh-NC ACHN, and Caki-1 stable transduction cells and performed sequencing at the transcriptional level (Fig. S9A). The differential mRNAs are presented as heatmaps in Fig. S9B. Further, bubble map, KEGG enrichment analysis, and enrichment chord plot suggested that the Wnt/β-catenin signaling pathway was significantly enriched (Fig. 8A and Fig. S9C, D). The results of IHC staining also showed that β-catenin expression was positively correlated with the tumor stage (Fig. S9E). Western blotting showed that overexpression of SLERCC could significantly increase the levels of UPF1 and decreased those of p-β-catenin, VEGFA, and BMI1, while knocking down SLERCC exerted the opposite effects (Fig. 8L, M). Subsequently, we performed rescue experiments in the PSPM-NPs 786-O cell line and sh-SLERCC#3 ACHN cell line to evaluate the effects of UPF1 on SLERCC-mediated functions. qRT-PCR analysis showed that knocking down UPF1 in PSPM-NPs 786-O cells could partially restored UPF1 expression, while overexpression of UPF1 in sh-SLERCC#3 ACHN cells, partially increased the levels of UPF1 (Fig. 8B, C). In addition, CCK8, EdU, wound healing, Transwell, angiogenesis, and tumorsphere assays, along with western blotting suggested that knocking down UPF1 could partially rescue the decrease in the tumor malignant behavior owing to SLERCC overexpression; conversely, overexpression of UPF1 partially inhibited the enhanced tumor malignant behaviors due to knocking down of SLERCC (Fig. 8D-K and Fig. S10A-D).

## Discussion

RCC is a common malignancy in the urological system, and its treatment, especially in metastatic cases, to date, poses a challenge for urologists [32]. Although TKI and ICI-based drugs have been developed for the treatment of metastatic RCC, the prognoses of patients with metastatic RCC remain poor, evidenced by the 5-year survival rates of less than 10% [33]. Given the low survival rate of patients with metastatic RCC, there is a need for developing targeted treatment strategies for patients with metastatic RCC [34, 35]. In the present study, we found that DNMT3A contributed significantly to the hypermethylation of SLERCC, leading to the downregulation of SLERCC expression in RCC, which in turn could accelerate the progression and metastasis in RCC by activating the Wnt/β-catenin signaling pathway. Loss and/or mutation of tumour-suppressor genes is a dominant force in tumour development and clinical resistance to a variety of therapies. Reversal of the phenotype induced by loss of tumour suppressors has long proven to be an elusive goal. Two major strategies have been employed for suppressor restoration, restoring a functional copy of a given tumour-suppressor gene via transfection; and the use of small-molecule agents to reactivate tumoursuppressor function via a conformational change in the mutated molecule. Therefore, we synthesized PSPM-NPs that could target the RCC cell surface molecule, MUC12, and the results demonstrated that plasmid-encapsulated NPs that target transmembrane metastasis markers may open up a new avenue for the treatment of RCC.

SLERCC was validated as a better prognostic biomarker in RCC clinical specimens. Analysis of clinicopathological parameters showed that lower SLERCC expression was associated with advanced tumor grade, stage, and poorer prognostic survival. In addition, the ROC curve had an AUC of 0.799 for SLERCC expression, which demonstrated the high accuracy of SLERCC in distinguishing RCC from normal tissues. Taken together, these results suggested that SLERCC is a valid tumor biomarker for RCC.

In this study, we also examined the biological functions of SLERCC in RCC. CCK8, EdU, wound healing, and Transwell assays showed that upregulation of SLERCC could significantly suppress the proliferation and invasion of RCC cells. Tube formation and tumorsphere formation assays showed that knocking down SLERCC could promote the angiogenic ability and stemness of RCC cells. Assays on xenograft animals, lung metastasis, and PDX models further confirmed that SLERCC upregulation inhibited tumor growth and metastasis. Although these results demonstrated that SLERCC played a role of tumor suppressor in RCC, the mechanism of SLERCC inactivation in RCC remains unknown.

Aberrant DNA methylation is a common epigenetic regulatory mechanism underlying tumors [36–38]. DNA methylation is mediated by DNMTs, including DNMT1, DNMT3A, and DNMT3B [39]. Here, we found that knocking down DNMT3A resulted in increased expression of SLERCC; moreover, the SLERCC promoter was hypermethylated in RCC. In addition, DNMT3A was recruited to the promoter region of SLERCC in RCC cells, which induced its hypermethylation, and the DNA methyltransferase inhibitor, 5-AZA, could rescue the reduced expression of SLERCC. Furthermore, mechanistically, SLERCC could directly bind to UPF1 and exert tumor-suppressive effects through the Wnt/β-catenin signaling pathway, thereby inhibiting the progression and metastasis of RCC.

The use of NPs as carriers for gene transfer is the future direction in the field of gene therapy for oncological diseases [40]. In recent years, the emergence of nanotechnology has provided opportunities for effective drug delivery to tumors [41]. Liposomes and PDA NPs are considered an attractive alternative to gold NPs in the field of nanomedicine [42–45]. Liposomes and PDA NPs have good biocompatibility and biodegradability [46, 47]. MUC12 is a membrane glycoprotein that is mainly localized to the cell membranes and is highly expressed on the surface of cells in colorectal cancer, hepatocellular carcinoma, and MUC12 is highly expressed in RCC, suggesting that MUC12 could also serve as a potential surface molecular marker for RCC cells [22, 23, 48]. Considering these findings, we proposed to rationally design liposomal PDA NPs to target MUC12 on the surface of RCC, and thus, deliver the plasmids encapsulated in the NPs to exert their intracellular functions. We developed a nanostructure with liposomes and PDA as carriers to deliver plasmids, which in turn could be used for therapeutic purposes in RCC.

Currently, the research on lncRNAs remains limited to the evaluation of underlying mechanisms and lacks further applications and treatment prospects. In this study, first, we demonstrated that SLERCC functions as a tumor suppressor and inhibits the progression and metastasis of RCC. Second, we found that DNMT3A was recruited at the promoter region of SLERCC in RCC cells, which in turn, induced its hypermethylation, eventually leading to downregulation of SLERCC expression. Finally, we synthesized liposomal PDA NPs encapsulating plasmid SLERCC which was capable of effectively and specifically targeting intracellular SLERCC. The results demonstrated that nanomaterials encapsulating plasmid targeting transmembrane transfer markers may open up a new avenue for therapy for RCC.

## Conclusions

To summarize, DNMT3A induced hypermethylation in the promoter region of SLERCC leading to its downregulation. SLERCC acts as a tumor suppressor lncRNA which inhibits RCC progression and metastasis through the Wnt/β-catenin signaling pathway during the progression of RCC. In addition, we constructed PSPM-NPs encapsulating SLERCC plasmids, which is expected to provide new insights for the development of future gene therapy-related drugs for the treatment of RCC.

## Supplementary Information

**Additional file 1. **

## Data Availability

The data that support the findings of this study are available from the corresponding author upon reasonable request.

## Abbreviations

lncRNAs: Long non-coding RNAs
ccRCC: Clear cell renal cell carcinomas
RCC: Renal cell carcinoma
PSPM-NPs: Plasmid-SLERCC@PDA@MUC12 nanoparticles
MUC12: Mucin 12
SLERCC: Specific Low Expression in RCC
OS: Overall survival
DFS: Disease-free survival
PDA: Polydopamine
AJCC: American Joint Committee on Cancer
TCGA: The Cancer Genome Atlas
FPKM: Fragments Per Kilobase Million
FDR: False discovery rate
qRT-PCR: Quantitative real-time polymerase chain reaction
CCK-8: Cell Counting Kit-8
OD: Optical density
FISH: Fluorescence in situ hybridization
5-AZA: 5′-Azacytidine
BSP: Bisulfite sequencing PCR
RIP: RNA immunoprecipitation
IHC: Immunohistochemistry
TEM: Transmission electron microscopy
STEM: Scanning transmission electron microscopy
EDS: Energy dispersive X-ray spectrometry
SDS-PAGE: Sodium dodecyl sulfate–polyacrylamide gel
AUC: Area under the curve
BUN: Blood urea nitrogen
ALT: Alanine aminotransferase
AST: Aspartate aminotransferase
DNMTs: DNA methyltransferases

## References

[1] Hsieh JJ, Purdue MP, Signoretti S, Swanton C, Albiges L, Schmidinger M (2017). Renal cell carcinoma. Nat Rev Dis Primers.

[2] Sung H, Ferlay J, Siegel RL, Laversanne M, Soerjomataram I, Jemal A (2021). Global cancer statistics 2020: GLOBOCAN estimates of incidence and mortality worldwide for 36 cancers in 185 countries. CA Cancer J Clin.

[3] Mao W, Wang K, Xu B, Zhang H, Sun S, Hu Q (2021). ciRS-7 is a prognostic biomarker and potential gene therapy target for renal cell carcinoma. Mol Cancer.

[4] Kotecha RR, Motzer RJ, Voss MH (2019). Towards individualized therapy for metastatic renal cell carcinoma. Nat Rev Clin Oncol.

[5] Lam JS, Belldegrun AS, Pantuck AJ (2006). Long-term outcomes of the surgical management of renal cell carcinoma. World J Urol.

[6] Li X, Wu Z, Fu X, Han W (2014). lncRNAs: insights into their function and mechanics in underlying disorders. Mutat Res Rev Mutat Res.

[7] Chen YF, Li YJ, Chou CH, Chiew MY, Huang HD, Ho JH (2020). Control of matrix stiffness promotes endodermal lineage specification by regulating SMAD2/3 via lncRNA LINC00458. Sci Adv..

[8] Guttman M, Rinn JL (2012). Modular regulatory principles of large non-coding RNAs. Nature.

[9] Balas MM, Hartwick EW, Barrington C, Roberts JT, Wu SK, Bettcher R, et al. Establishing RNA-RNA interactions remodels lncRNA structure and promotes PRC2 activity. Sci Adv. 2021;7(16):eabc9191. 10.1126/sciadv.abc9191 PMC8046370 33853770

[10] Martens-Uzunova ES, Bottcher R, Croce CM, Jenster G, Visakorpi T, Calin GA (2014). Long noncoding RNA in prostate, bladder, and kidney cancer. Eur Urol.

[11] Kulkarni P, Dasgupta P, Hashimoto Y, Shiina M, Shahryari V, Tabatabai ZL (2021). A lncRNA TCL6-miR-155 interaction regulates the Src-Akt-EMT network to mediate kidney cancer progression and metastasis. Cancer Res.

[12] Braga EA, Fridman MV, Filippova EA, Loginov VI, Pronina IV, Burdennyy AM, et al. LncRNAs in the Regulation of Genes and Signaling Pathways through miRNA-Mediated and Other Mechanisms in Clear Cell Renal Cell Carcinoma. Int J Mol Sci. 2021;22(20):11193. 10.3390/ijms222011193 PMC8539140 34681854

[13] Lorenzen JM, Thum T (2016). Long noncoding RNAs in kidney and cardiovascular diseases. Nat Rev Nephrol.

[14] Zhai W, Sun Y, Guo C, Hu G, Wang M, Zheng J (2017). LncRNA-SARCC suppresses renal cell carcinoma (RCC) progression via altering the androgen receptor(AR)/miRNA-143-3p signals. Cell Death Differ.

[15] Hirata H, Hinoda Y, Shahryari V, Deng G, Nakajima K, Tabatabai ZL (2015). Long Noncoding RNA MALAT1 promotes aggressive renal cell carcinoma through Ezh2 and Interacts with miR-205. Cancer Res.

[16] Barth DA, Juracek J, Slaby O, Pichler M, Calin GA. lncRNA and Mechanisms of Drug Resistance in Cancers of the Genitourinary System. Cancers (Basel). 2020;12(8):2148. 10.3390/cancers12082148 PMC7463785 32756406

[17] Liu X, Hao Y, Yu W, Yang X, Luo X, Zhao J (2018). Long non-coding rna emergence during renal cell carcinoma tumorigenesis. Cell Physiol Biochem.

[18] Li M, Wang Y, Cheng L, Niu W, Zhao G, Raju JK (2017). Long non-coding RNAs in renal cell carcinoma: a systematic review and clinical implications. Oncotarget.

[19] Matsuyama T, Ishikawa T, Mogushi K, Yoshida T, Iida S, Uetake H (2010). MUC12 mRNA expression is an independent marker of prognosis in stage II and stage III colorectal cancer. Int J Cancer.

[20] Pham E, Friedrich M, Aeffner F, Lutteropp M, Mariano NF, Deegen P (2021). Preclinical assessment of a MUC12-targeted BiTE (bispecific t-cell engager) molecule. Mol Cancer Ther.

[21] Byrd JC, Bresalier RS (2004). Mucins and mucin binding proteins in colorectal cancer. Cancer Metastasis Rev.

[22] Zhang B, Chu W, Wen F, Zhang L, Sun L, Hu B (2020). Dysregulation of long non-coding RNAs and mRNAs in plasma of clear cell renal cell carcinoma patients using microarray and bioinformatic analysis. Front Oncol.

[23] Gao SL, Yin R, Zhang LF, Wang SM, Chen JS, Wu XY (2020). The oncogenic role of MUC12 in RCC progression depends on c-Jun/TGF-beta signalling. J Cell Mol Med.

[24] d'Ischia M, Napolitano A, Ball V, Chen CT, Buehler MJ (2014). Polydopamine and eumelanin: from structure-property relationships to a unified tailoring strategy. Acc Chem Res.

[25] Lee Haesung A, Do Minjae Seo (2017). Polydopamine coating in organic solvent for material-independent immobilization of water-insoluble molecules and avoidance of substrate hydrolysi.

[26] Li Z, Zhang X, Wang S, Yang Y, Qin B, Wang K (2016). Polydopamine coated shape memory polymer: enabling light triggered shape recovery, light controlled shape reprogramming and surface functionalization. Chem Sci.

[27] Li W, Yang FQ, Sun CM, Huang JH, Zhang HM, Li X (2020). circPRRC2A promotes angiogenesis and metastasis through epithelial-mesenchymal transition and upregulates TRPM3 in renal cell carcinoma. Theranostics.

[28] Mao W, Wang K, Sun S, Wu J, Chen M, Geng J (2021). ID2 Inhibits bladder cancer progression and metastasis via PI3K/AKT signaling pathway. Front Cell Dev Biol.

[29] Wang K, Gu Y, Ni J, Zhang H, Wang Y, Zhang Y (2022). Noncoding-RNA mediated high expression of zinc finger protein 268 suppresses clear cell renal cell carcinoma progression by promoting apoptosis and regulating immune cell infiltration. Bioengineered.

[30] Li Y, Liu HT, Chen X, Wang YW, Tian YR, Ma RR (2022). Aberrant promoter hypermethylation inhibits RGMA expression and contributes to tumor progression in breast cancer. Oncogene.

[31] Zhao ZW, Lian WJ, Chen GQ, Zhou HY, Wang GM, Cao X (2012). Decreased expression of repulsive guidance molecule member A by DNA methylation in colorectal cancer is related to tumor progression. Oncol Rep.

[32] Li P, Wong YN, Armstrong K, Haas N, Subedi P, Davis-Cerone M (2016). Survival among patients with advanced renal cell carcinoma in the pretargeted versus targeted therapy eras. Cancer Med.

[33] Albiges L, Powles T, Staehler M, Bensalah K, Giles RH, Hora M (2019). Updated European association of urology guidelines on renal cell carcinoma: immune checkpoint inhibition is the new backbone in first-line treatment of metastatic clear-cell renal cell carcinoma. Eur Urol.

[34] Medina Lopez RA, Rivero Belenchon I, Mazuecos-Quiros J, Congregado-Ruiz CB, Counago F (2022). Update on the treatment of metastatic renal cell carcinoma. World J Clin Oncol.

[35] Roberto M, Botticelli A, Panebianco M, Aschelter AM, Gelibter A, Ciccarese C (2021). Metastatic renal cell carcinoma management: from molecular mechanism to clinical practice. Front Oncol.

[36] Klutstein M, Nejman D, Greenfield R, Cedar H (2016). DNA methylation in cancer and aging. Cancer Res.

[37] Esteller M (2005). Aberrant DNA methylation as a cancer-inducing mechanism. Annu Rev Pharmacol Toxicol.

[38] Saghafinia S, Mina M, Riggi N, Hanahan D, Ciriello G (2018). Pan-Cancer Landscape of Aberrant DNA Methylation across Human Tumors. Cell Rep.

[39] Subramaniam D, Thombre R, Dhar A, Anant S (2014). DNA methyltransferases: a novel target for prevention and therapy. Front Oncol.

[40] Forbes DC, Peppas NA (2012). Oral delivery of small RNA and DNA. J Control Release.

[41] Gao Z, Zhang L, Sun Y (2012). Nanotechnology applied to overcome tumor drug resistance. J Control Release.

[42] Cheng W, Zeng X, Chen H, Li Z, Zeng W, Mei L (2019). Versatile polydopamine platforms: synthesis and promising applications for surface modification and advanced nanomedicine. ACS Nano.

[43] Gholami Derami H, Gupta P, Weng KC, Seth A, Gupta R, Silva JR (2021). Reversible photothermal modulation of electrical activity of excitable cells using polydopamine nanoparticles. Adv Mater.

[44] Mrowczynski R (2018). Polydopamine-based multifunctional (nano)materials for cancer therapy. ACS Appl Mater Interfaces.

[45] Schanze KS, Lee H, Messersmith PB (2018). Ten years of polydopamine: current status and future directions. ACS Appl Mater Interfaces.

[46] Seth A, Gholami Derami H, Gupta P, Wang Z, Rathi P, Gupta R (2020). Polydopamine-mesoporous silica core-shell nanoparticles for combined photothermal immunotherapy. ACS Appl Mater Interfaces.

[47] Bi D, Zhao L, Li H, Guo Y, Wang X, Han M (2019). A comparative study of polydopamine modified and conventional chemical synthesis method in doxorubicin liposomes form the aspect of tumor targeted therapy. Int J Pharm.

[48] Huang H, Hu Y, Guo L, Wen Z (2021). Integrated bioinformatics analyses of key genes involved in hepatocellular carcinoma immunosuppression. Oncol Lett.

