# Supplementary material for: Transfection with Plasmid-Encoding lncRNA-SLERCC nanoparticle-mediated delivery suppressed tumor progression in renal cell carcinoma
Source: J Exp Clin Cancer Res. 2022 Aug 19;41:252. doi: 10.1186/s13046-022-02467-2 (PMC9389749; doi:10.1186/s13046-022-02467-2)
Supplement: Supplementary file 1 — Additional file 1. [file 13046_2022_2467_MOESM1_ESM.docx]

Supporting Information

**Transfection with Plasmid-Encoding lncRNA-SLERCC nanoparticle-mediated delivery suppressed tumor progression in renal cell carcinoma**

Weipu Mao, Keyi Wang, Wentao Zhang, Shuqiu Chen, Jinbo Xie, Zongtai Zheng, Xue Li, Ning Zhang, Yuanyuan Zhang, Haimin Zhang, Bo Peng, Xudong Yao, Jianping Che^*^, Junhua Zheng^*^, Ming Chen^*^, Wei Li^*^

**Supplementary figures and tables**


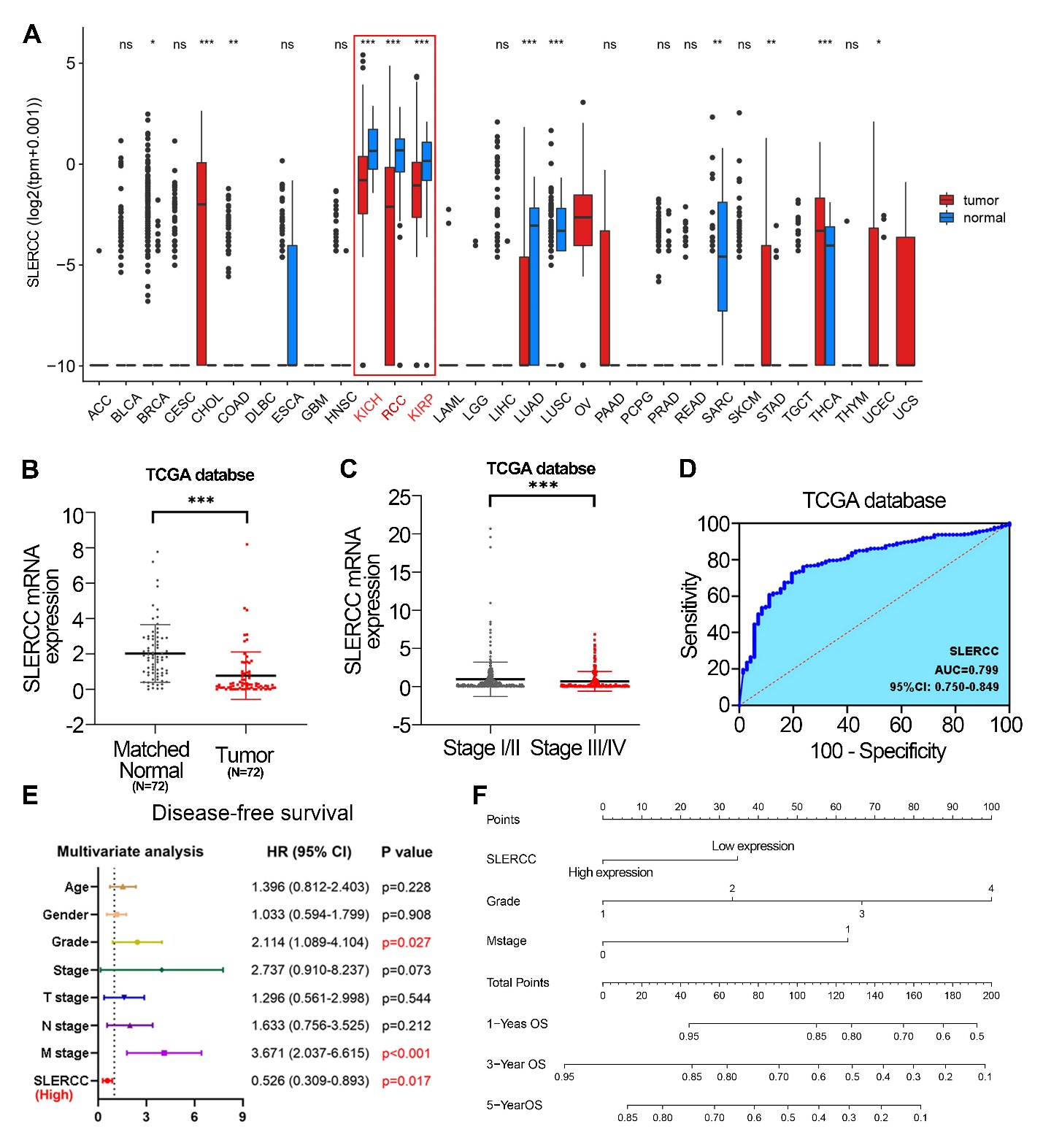


**Figure S1** **Construction of SLERCC-based prognostic nomogram for overall survival of patients with RCC** **in TCGA dataset.** (A) Expression of SLERCC in different cancer types. (B) Expression of SLERCC in tumor and matched normal tissues. (C) Relative levels of expression of SLERCC in different stage subgroups. (D) ROC curve shows the efficiency of SLERCC expression to distinguish KIRC from non-tumor tissues. (E) Multivariate cox regression analyses of DFS for SLERCC expression with disease-free survival (DFS). (F) Overall survival (OS) nomogram predicted the 1-, 3-, and 5-year survival. (*p < 0.05, **p < 0.01, ***p < 0.001).

Abbreviations: ACC, Adrenocortical carcinoma; BLCA, Bladder urothelial carcinoma; BRCA, Breast invasive carcinoma; CESC, Cervical squamous cell carcinoma and endocervical adenocarcinoma; CHOL, Cholangiocarcinoma; COAD, Colon adenocarcinoma; DLBC, Lymphoid neoplasm diffuse large B-cell lymphoma; ESCA, Esophageal carcinoma; GBM, Glioblastoma multiforme; HNSC, Head and neck squamous cell carcinoma; KICH, Kidney Chromophobe; RCC, Renal cell carcinoma; KIRP, Kidney renal papillary cell carcinoma; LAML, Acute myeloid leukemia; LGG, Brain lower grade glioma; LIHC, Liver hepatocellular carcinoma; LUAD, Lung adenocarcinoma; LUSC, Lung squamous cell carcinoma; OV, Ovarian serous cystadenocarcinoma; PAAD, Pancreatic adenocarcinoma; PCPG, Pheochromocytoma and paraganglioma; PRAD, Prostate adenocarcinoma; READ, Rectum adenocarcinoma; SARC, Sarcoma; SKCM, Skin cutaneous melanoma; STAD, Stomach adenocarcinoma; TGCT, Testicular germ cell tumors; THCA, Thyroid carcinoma; THYM, Thymoma; UCEC, Uterine corpus endometrial carcinoma; UCS, Uterine carcinosarcoma

**
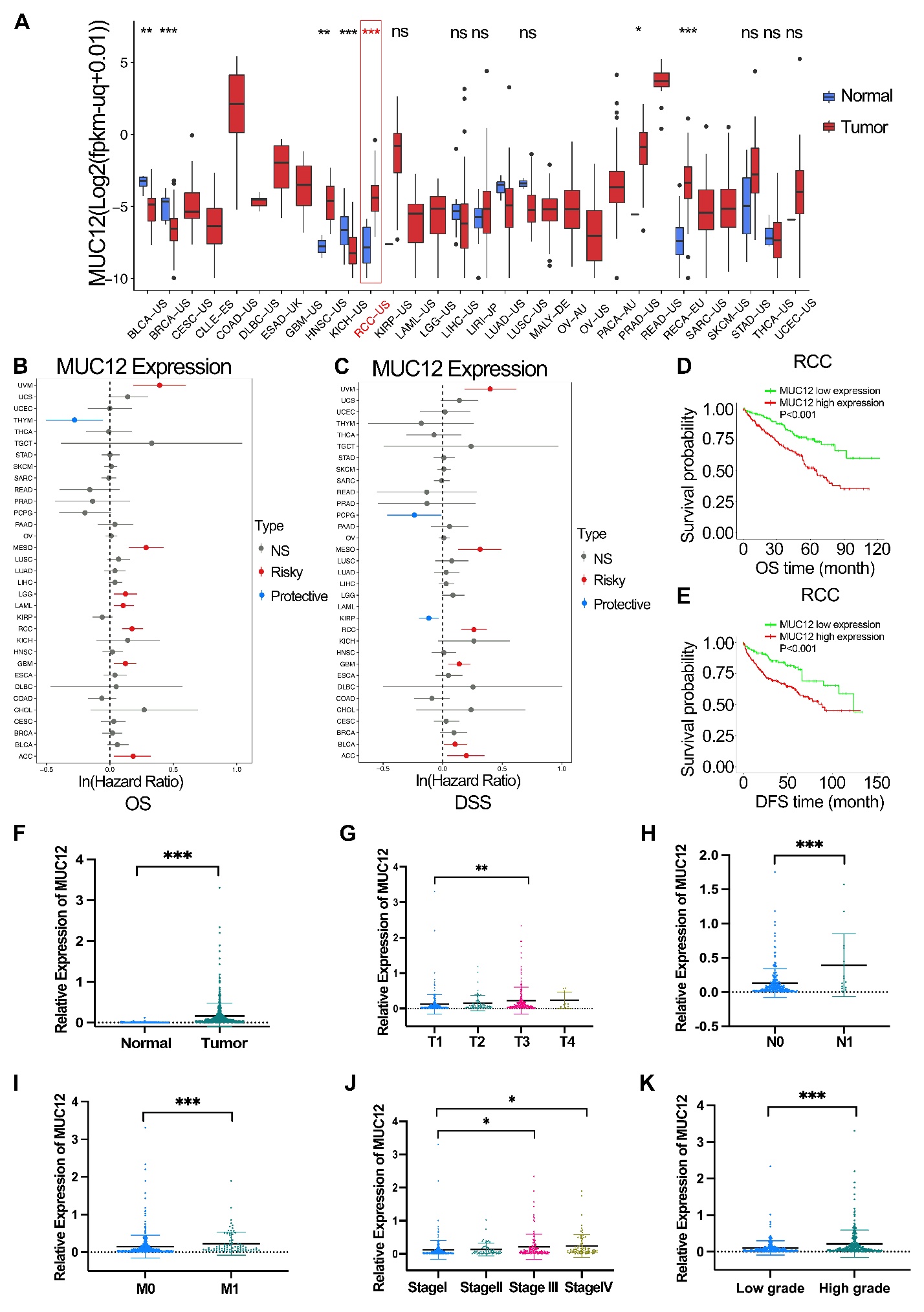
Figure S2** **MUC12 is upregulated in RCC tissues** **in TCGA dataset**. (A) Expression of MUC12 in different cancer types. (B, C) Multivariate analysis of OS and DFS of MUC12 expression in different tumor types. (D, E) Overall survival (D) and disease-free survival (E) curves for RCC patients with low and high SLERCC expression. (F) Expression of MUC12 in normal and tumor tissues. (G-H) Relative expression levels of SLERCC in tissue samples for T-stage (G), N-stage (H), M-stage (I), tumor stage (J) and tumor grade (K). (*p < 0.05, **p < 0.01, ***p < 0.001).

Abbreviations: BLCA-US, Bladder urothelial carcinoma-TCGA, US; BRCA-US, Breast invasive carcinoma-TCGA, US; CESC-US, Cervical squamous cell carcinoma and endocervical adenocarcinoma-TCGA, US; CLLE-ES, Chronic Lymphocytic Leukemia-ES; COAD-US, Colon adenocarcinoma-TCGA, US; DLBC-US, Lymphoid neoplasm diffuse large B-cell lymphoma-TCGA, US; ESAD-UK, Esophageal Adenocarcinoma-UK; GBM-US, Glioblastoma multiforme-TCGA, US; HNSC-US, Head and neck squamous cell carcinoma-TCGA, US; KICH-US, Kidney Chromophobe-TCGA, US; RCC-US, Renal cell carcinoma-TCGA, US; KIRP-US, Kidney renal papillary cell carcinoma-TCGA, US; LAML-US, Acute myeloid leukemia-TCGA, US; LGG-US, Brain lower grade glioma-TCGA, US; LIHC-US, Liver hepatocellular carcinoma-TCGA, US; LIRI-JP, Liver Cancer-RIKEN, JP; LUAD-US, Lung adenocarcinoma-TCGA, US; LUSC-US, Lung squamous cell carcinoma-TCGA, US; MALY-DE. Malignant Lymphoma-DE; OV-AU, Ovarian serous cystadenocarcinoma-AU; OV-US, Ovarian serous cystadenocarcinoma-TCGA, US; PACA-AU, Pancreatic Cancer-AU; PRAD-US, Pancreatic adenocarcinoma-TCGA, US; READ-US, Rectum adenocarcinoma-TCGA, US; RECA-EU, Renal cell cancer-EU/FR; SARC-US, Sarcoma-TCGA, US; SKCM-US, Skin cutaneous melanoma-TCGA, US; STAD-US, Stomach adenocarcinoma-TCGA, US; THCA-US, Thyroid carcinoma-TCGA, US; UCEC-US, Uterine corpus endometrial carcinoma-TCGA, US


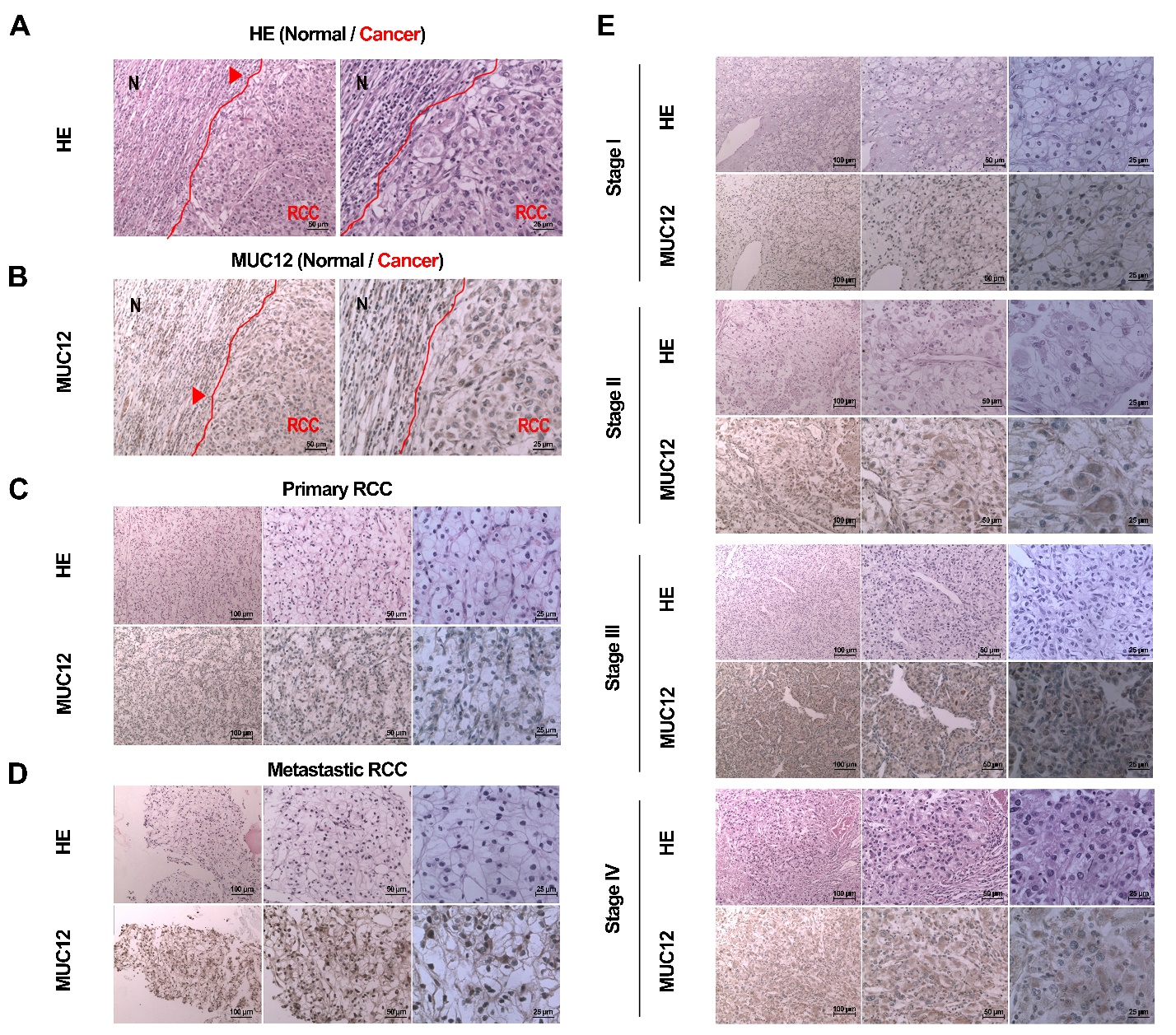


**Figure S3 IHC and HE staining of MUC12 in RCC tissues.** (A) HE staining for MUC12 in normal and cancer tissue. (B) IHC staining for MUC12 in normal and cancer tissue. (C) IHC and HE staining for MUC12 in primary RCC tissues. (D) IHC and HE staining for MUC12 in metastatic RCC tissues. (E) IHC and HE staining for MUC12 in different stage tissues.

Abbreviations: IHC, immunohistochemistry; RCC, Renal cell carcinoma.


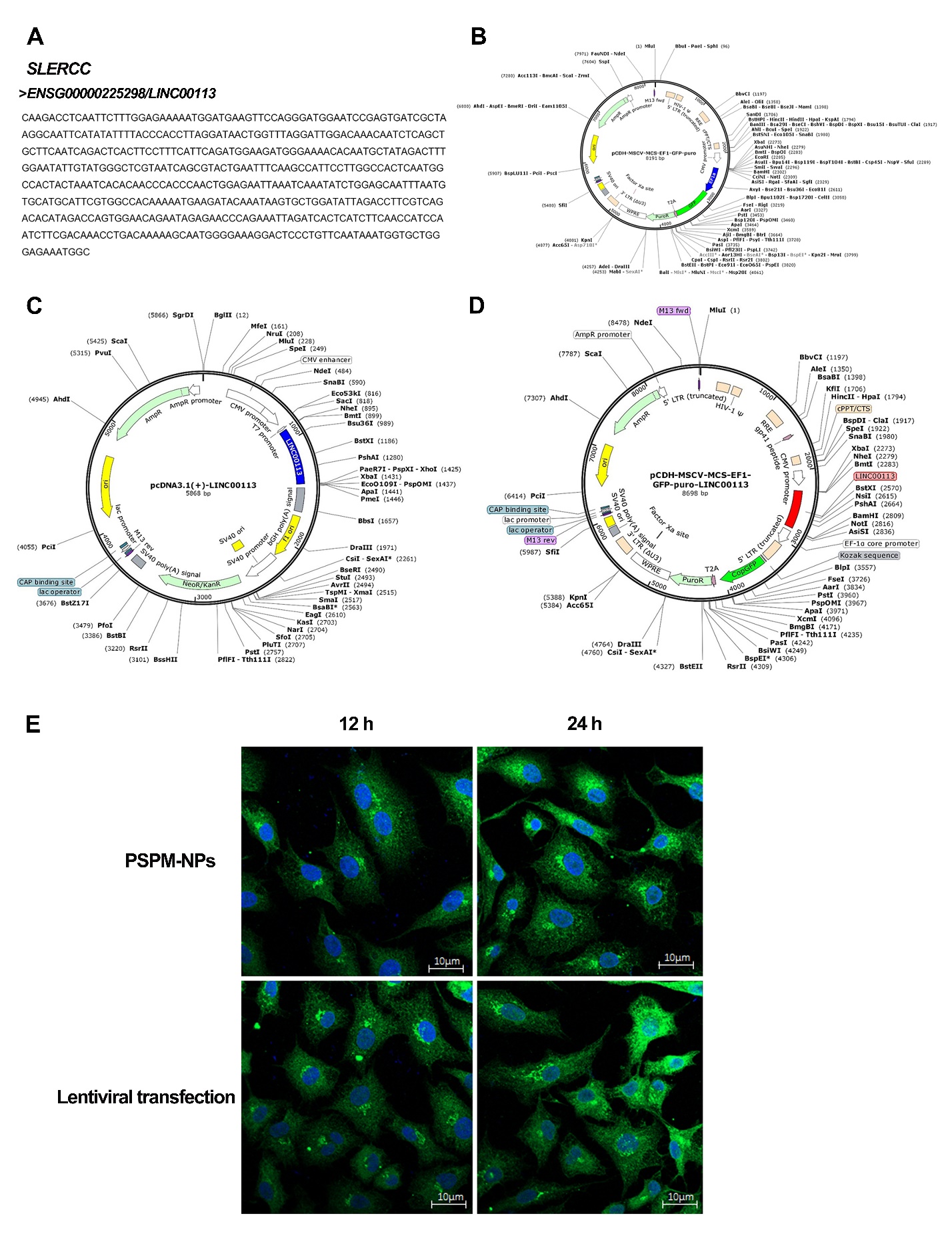


**Figure S4** **Construction and transfection effect of SLERCC overexpression vector.** (A) Transient plasmid of SLERCC. (B) Full-length sequence information of SLERCC. (C) Overexpression empty vector. (D) SLERCC complete overexpression lentiviral construct. (E) Comparison of transfection effects of PSPM-NPs and conventional SLERCC overexpression lentivirus.

Abbreviations: PSPM-NPs, Plasmid-SLERCC@PDA@MUC12 nanoparticles.


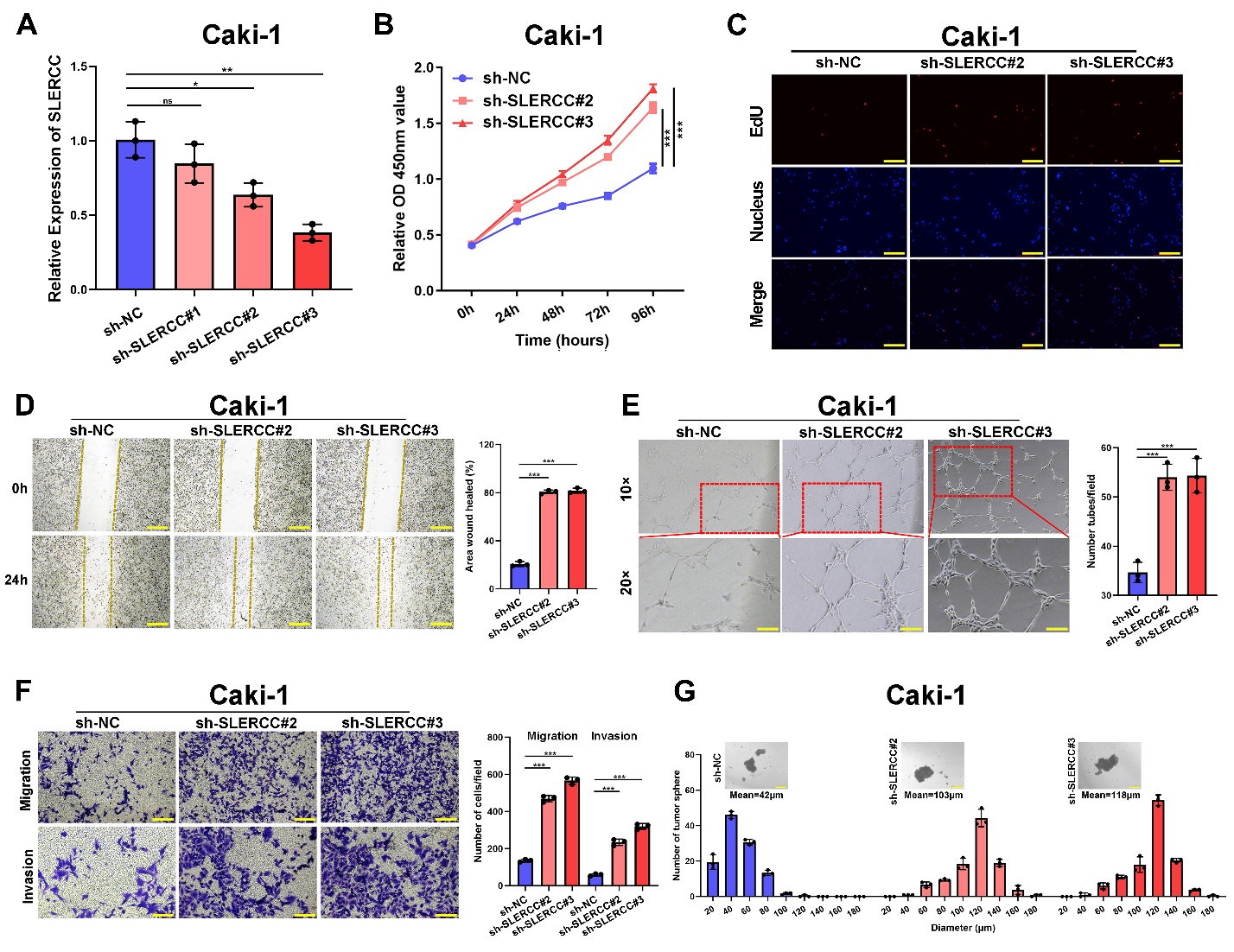


**Figure S5** **SLERCC as a tumor suppressor inhibits the progression of RCC cells *in vitro*.** (A) Relative expression of SLERCC in Caki-1 cell lines transfected with sh-NC, sh-SLERCC#1, sh-SLERCC#2, or sh-SLERCC#3 constructs. (B) Growth curves of Caki-1 cells were measured after transfection with indicated vectors by CCK-8 assays. (C) Growth curves of Caki-1 cells transfected with indicated vectors assessed by EdU assays. (D) The migratory capacity of Caki-1 cells transfected with indicated vectors assessed by the wound healing assay. (E) Cell migration and invasion of Caki-1 cells transfected with indicated vectors assessed by Transwell migration and Matrigel invasion assays. (F) The angiogenic capacity of Caki-1 cells transfected with indicated vectors assessed by tube formation assay. (G) Stemness of Caki-1 cells transfected with indicated vectors assessed by the tumorsphere assay. (*p < 0.05, **p < 0.01, ***p < 0.001).

Abbreviations: RCC, Renal cell carcinoma.


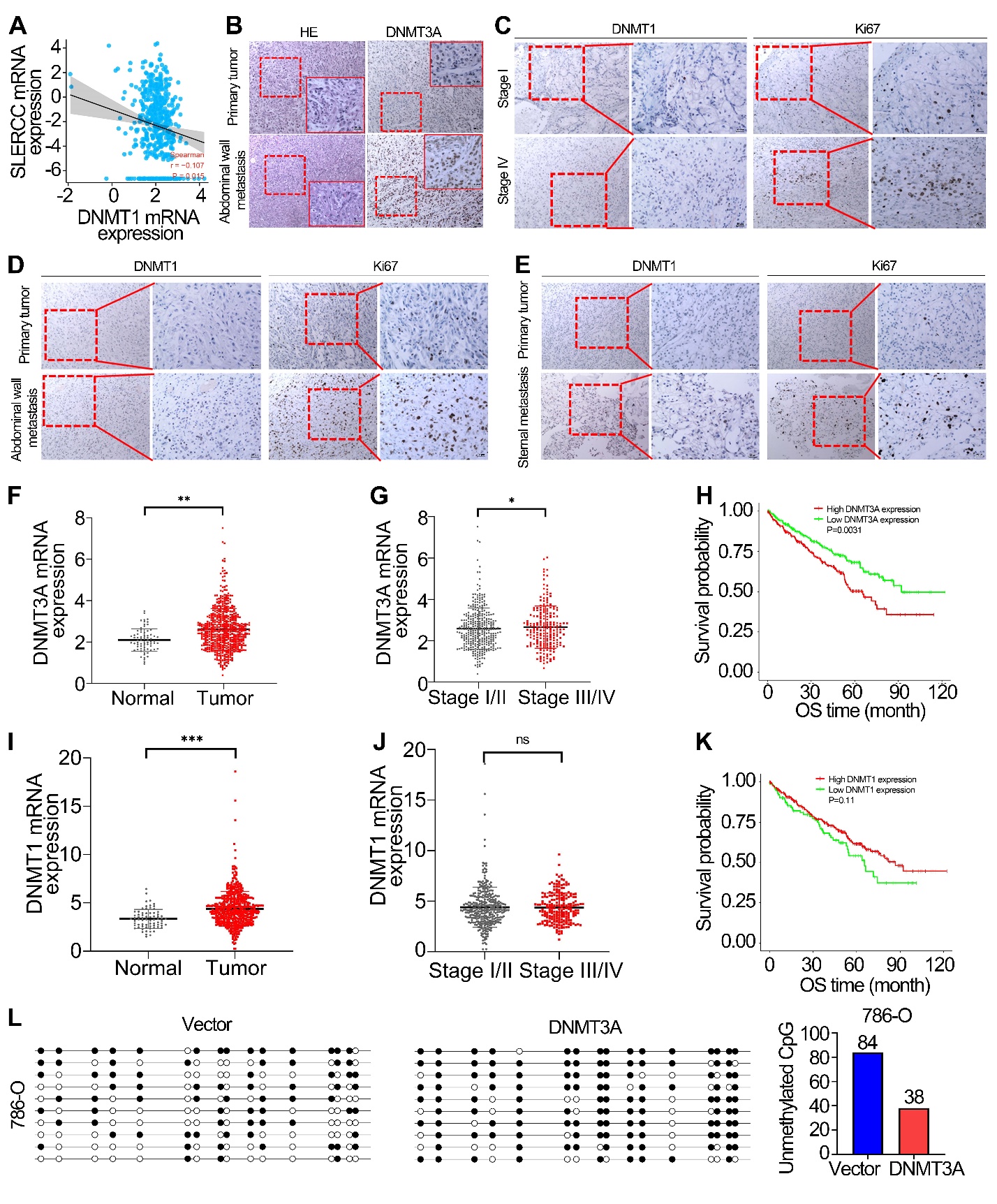
**Figure** **S6** **DNMT3A is upregulated in RCC tissues.** (A) Relationship between DNMT1 and SLERCC in the TCGA dataset. (B) IHC staining for DNMT3A in primary carcinoma and abdominal wall metastatic tissues. (C-E) IHC staining for DNMT1 and Ki67 in stages I and IV, primary carcinoma, sternal metastatic, and abdominal wall metastatic tissues. (F) Expression of DNMT3A in normal (n = 72) and tumor (n = 539) tissues in TCGA dataset. (G) Relative levels of expression of DNMT3A in different stage subgroups. (H) Overall survival curve for RCC patients with low and high DNMT3A expression. (I) Expression of DNMT1 in normal and tumor tissues in TCGA dataset. (J) Relative levels of expression of DNMT1 in different stage subgroups. (K) Overall survival curve for RCC patients with low and high DNMT1 expression. (L) BSP assay detects DNA methylation levels of DNMT3A at CpG islands in the SLERCC promoter region in 786-O cells. (*p < 0.05, **p < 0.01, ***p < 0.001).

Abbreviations: IHC, immunohistochemistry; RCC, Renal cell carcinoma.


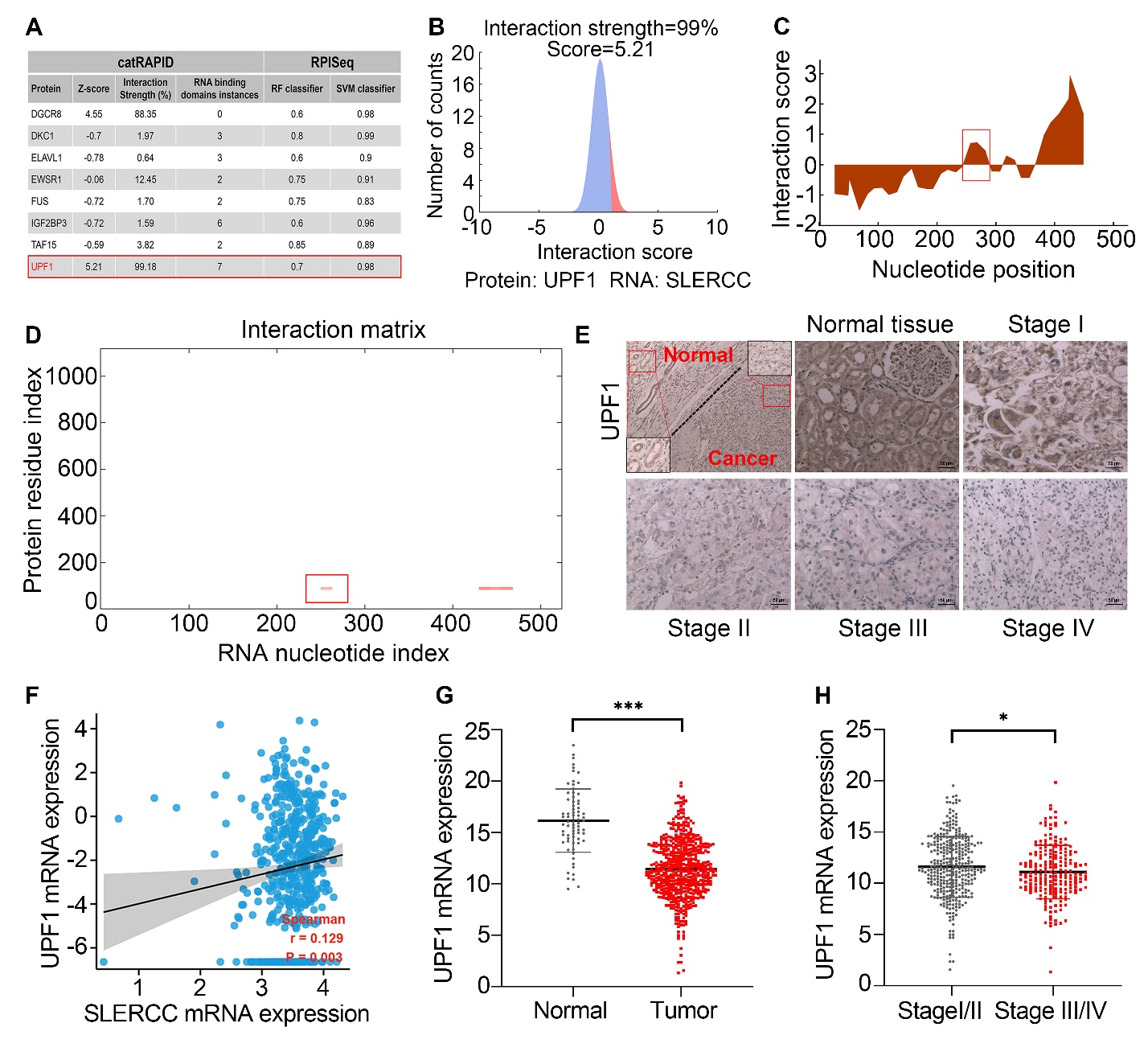


**Figure S7** **Software-based prediction of the putative proteins that directly bind to SLERCC.** (A) catRAPID and RPISeq analysis for evaluation of scores of the eight proteins. (B) catRAPID analysis for evaluating the interaction strength and score between SLERCC and UPF1. (C, D) The binding sites of UPF1 in the sequences of SLERCC as predicted using the catRAPID website. (E) IHC staining for UPF1 in normal tissues and tissues of different tumor stages. (F) Relationship between SLERCC and UPF1 in TCGA dataset. (G) Expression of UPF1 in (n = 72) and tumor (n = 539) tissues of TCGA dataset. (H) Relative levels of expression of UPF1 in different stage subgroups. (*p < 0.05, ***p < 0.001)

Abbreviations: IHC, immunohistochemistry.


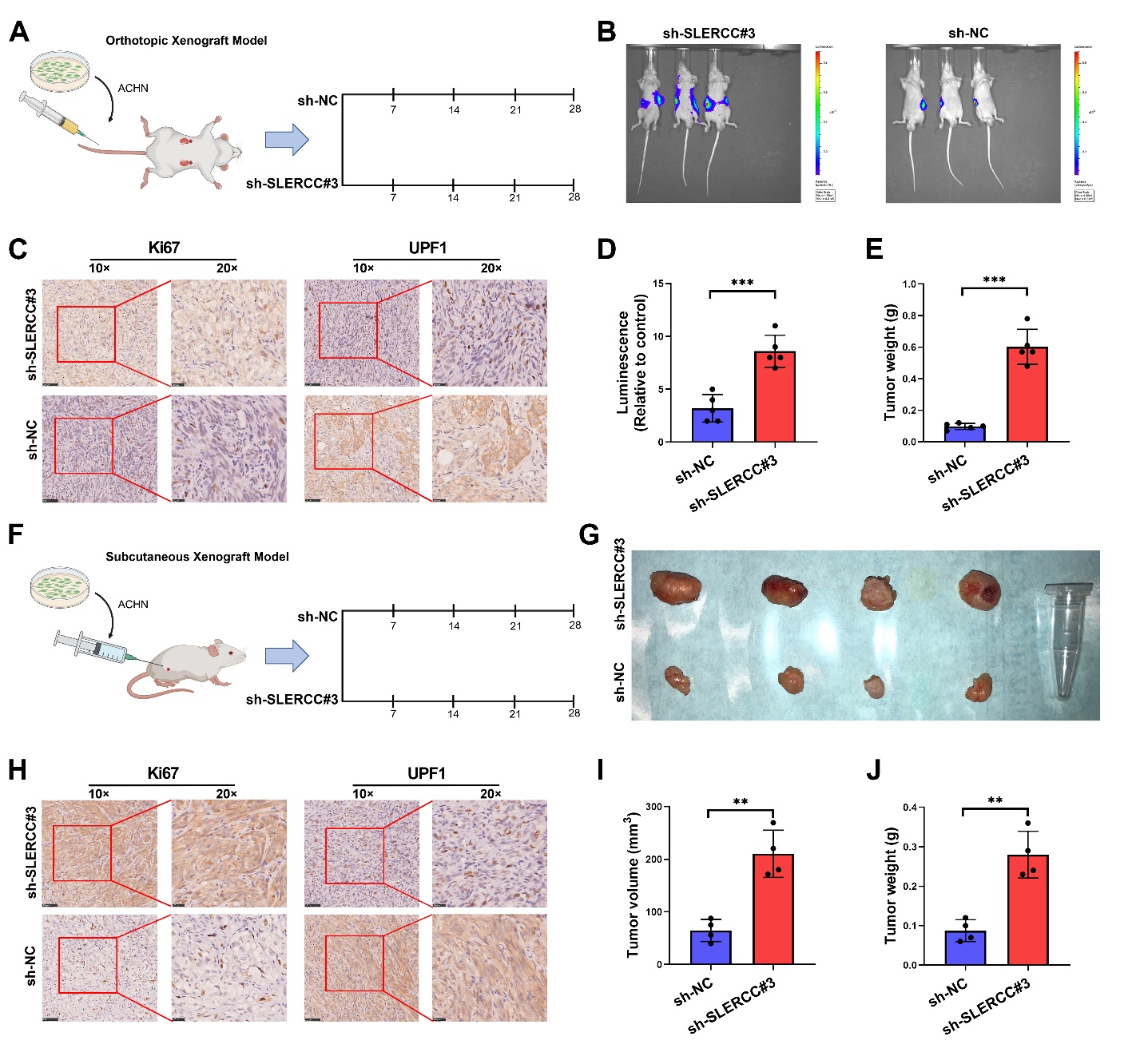


**Figure S8** **SLERCC knockdown accelerates RCC progression and metastasis *in vivo***. (A, B) Representative bioluminescence images of nude mice with orthotopically implanted sh-SLERCC#3 or sh-NC expressing ACHN cells. (C) Representative IHC images of subcutaneous xenograft model. (D, E) Lumineacence (D) and tumor weight (E) in the orthotopic xenograft model. (F, G) Representative bioluminescence images of nude mice with subcutaneou implanted sh-SLERCC#3 or sh-NC expressing ACHN cells. (H) Representative IHC images of subcutaneous xenograft model. (I, J) Tumor volume (I) and tumor weight (J) in the subcutaneous xenograft model. (**p < 0.01, ***p < 0.001).

Abbreviations: IHC, immunohistochemistry; RCC, Renal cell carcinoma.


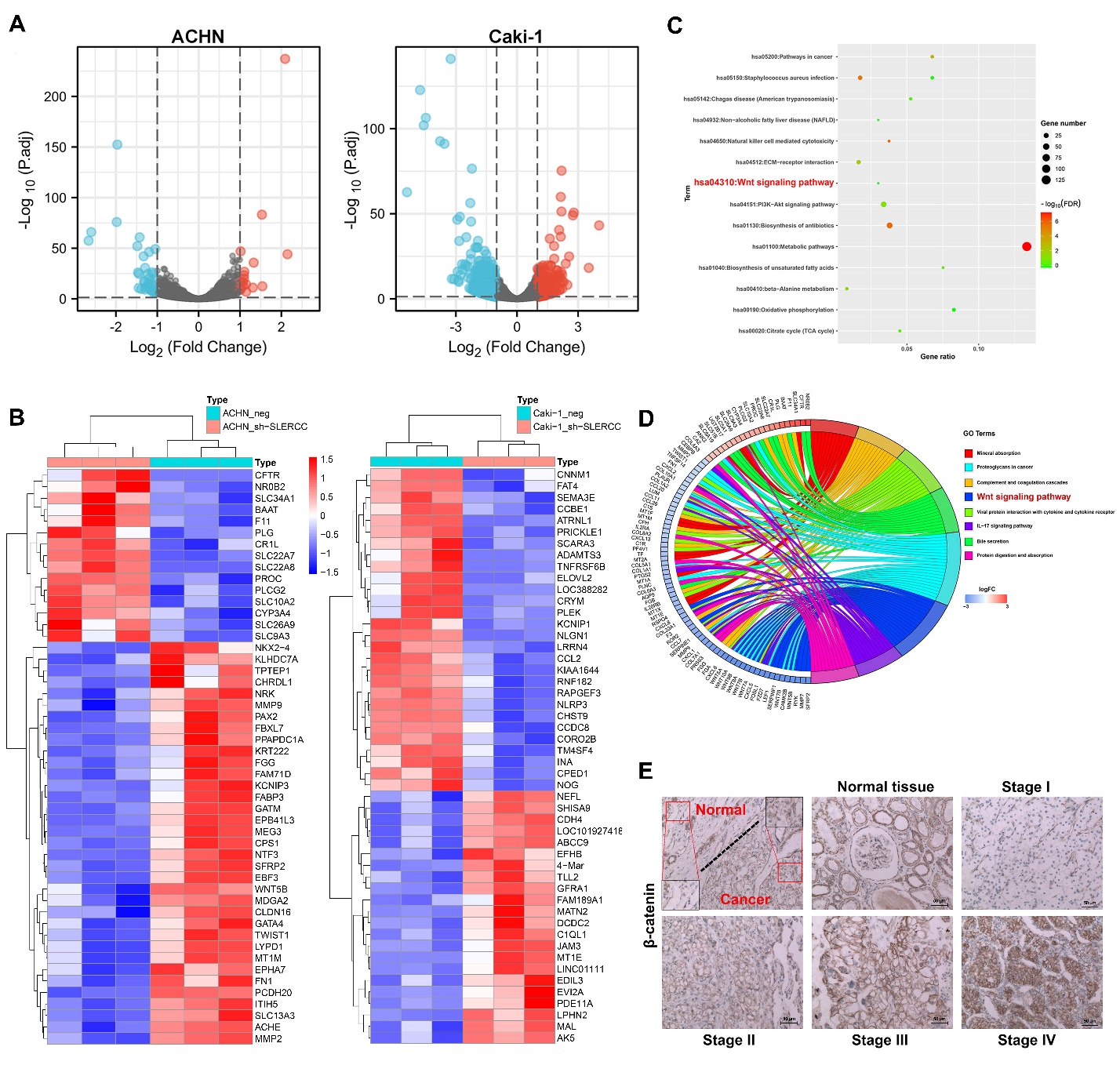


**Figure S9 Wnt/β-catenin signaling pathway is the downstream pathway of SLERCC**. (A) Volcano plot of differential genes in sh-SLERCC#3, sh-NC ACHN, and Caki-1 stably transduced cells. (B) The heatmap of differentially expressed genes. (C) Bubble map for differentially expressed genes. (D) Enrichment chord plot for different genes. (E) IHC staining for β-catenin in normal tissues and tissues at different tumor stages.

Abbreviations: IHC, immunohistochemistry.

**
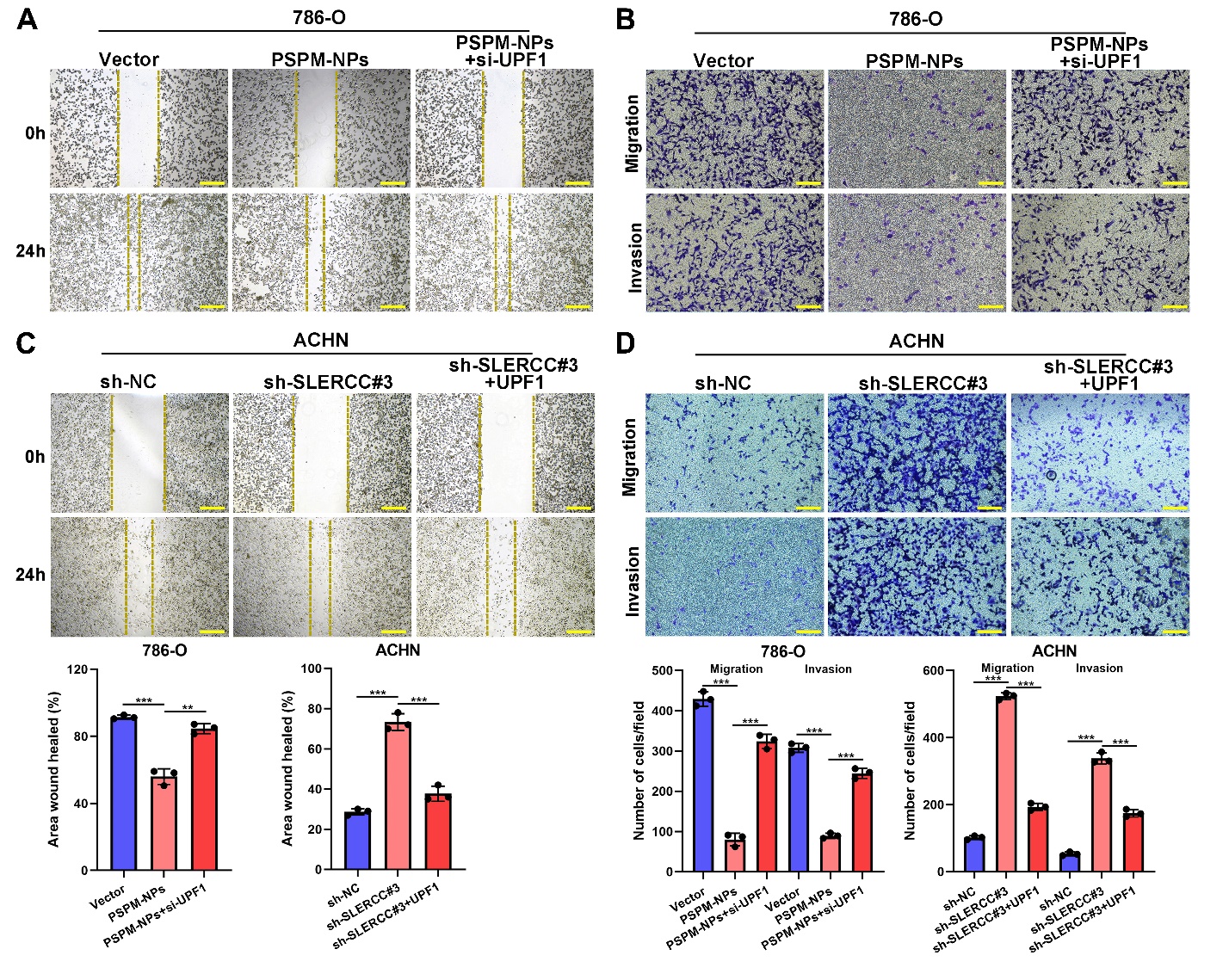
**

**Figure S10** **UPF1 rescues SLERCC-mediated function**. (A, C) The rescue effects of UPF1 on the migration of SLERCC expressing 786-O (A) and ACHN (C) cells as assessed by wound healing assay. (B, D) The rescue effects of UPF1 on the cell migration and invasion of SLERCC expressing 786-O (B) and ACHN (D) cells as assessed by Transwell migration and Matrigel invasion assays. (**p < 0.01, ***p < 0.001).

**Table S1** Characteristics of patients with RCC and associations with clinicopathologic variables.

| **Variable** | **Total (%)** | **Variable** | **Total (%)** |
| --- | --- | --- | --- |
| **Gender** |  | **Fuhrman grade** |  |
| Male | 55 (61.1) | 1-2 | 51 (56.7) |
| Female | 35 (38.9) | 3 | 28 (31.1) |
| **Age at surgery (years)** |  | 4 | 11 (12.2) |
| ≤45 | 5 (5.6) | **AJCC clinical stage** |  |
| >45, ≤55 | 32 (35.6) | I | 24 (26.7) |
| >55 | 53 (58.9) | II | 31 (34.4) |
| **BMI** |  | III | 15 (16.7) |
| <25 | 56 (62.2) | IV | 20 (22.2) |
| ≥25 | 34 (37.8) | **Metastatic status** |  |
| **Maximum tumor size (cm)** |  | NM | 69 (76.7) |
| ≤4 | 27 (30.0) | LM | 10 (11.1) |
| >4, ≤7 | 35 (38.9) | DM | 11 (12.2) |
| >7, ≤10 | 21 (23.3) | **T stage** |  |
| >10 | 7 (7.8) | T1 | 24 (26.7) |
| **Renal capsule invasion** |  | T2 | 33 (36.7) |
| No | 62 (68.9) | T3 | 13 (14.4) |
| Yes | 28 (31.1) | T4 | 20 (22.2) |

**Abbreviation:** BMI=body mass index; DM=tumours involving distant metastases; LM=tumours involving lymphatic metastases; NM=tumours involving non-metastasis; RCC=renal cell carcinoma.

**Table S2** PCR primer, siRNA and probe sequence.

| **Primes** | **5’-3’ sequence** |
| --- | --- |
| **Primers for PCR** |  |
| SLERCC-forward | TAATCAGCGTACTGAATTTCAAGC |
| SLERCC -reverse | TCTCCAGTTGGGTGGGTTGT |
| UPF1-forward | TCACGGCACAGCAGATCAACAAG |
| UPF1-reverse | CGGCTTCTCCAGGTCCTCCAG |
| GAPDH-forward | CAGGAGGCATTGCTGATGAT |
| GAPDH-reverse | GAAGGCTGGGGCTCATTT |
| U6-forward | CAAATTCGTGAAGCGTTCCATAT |
| U6-reverse | GCTTCACGAATTTGCGTGTCATCCTTGC |
| **siRNAs** |  |
| si-SLERCC#1-Sense | AGUGGAACAGAAUAGAGAACC |
| si-SLERCC#1-Antisense | UUCUCUAUUCUGUUCCACUGG |
| si-SLERCC#2-Sense | GAAUCCGAGUGAUCGCUAAGG |
| si-SLERCC#2-Antisense | UUAGCGAUCACUCGGAUUCCA |
| si-SLERCC#3-Sense | GCUGCUUCAAUCAGACUCACU |
| si-SLERCC#3-Antisense | UGAGUCUGAUUGAAGCAGCUG |
| sh-SLERCC#1 | GAAAGGACTCCCTGTTCAA |
| sh-SLERCC#2 | GAATCCGAGTGATCGCTAA |
| sh-SLERCC#3 | GCTGCTTCAATCAGACTCA |
| **RNA pull-down probes** |  |
| Probe 1 | GGTGGGTAAAATATATGAATTGCCTTA |
| Probe 2 | CAGTACGCTGATTACGAGCCCATACAATA |
| Probe 3 | GATGAGTGATCTAATTTCTGGGTTCTCTA |
| SLERCC-probe | CAGTACGCTGATTACGAGCCCATACAAT |
| FITC probe | CTTCGTCATCTCCCGAGGATGGAGCGTTCGGGGCCCGTGAACGCTC |
| cy3 probe | TTGTTTGTTGCGTCTGCCTCTCT |

**Table S3** Antibodies list.

| **Name** | **Company** | **Number** |
| --- | --- | --- |
| UPF1 | Abcam | Ab109363 |
| β-catenin | Abcam | Ab32572 |
| p-β-catenin | Abcam | Ab75777 |
| VEGFA | Abcam | Ab46154 |
| BMI1 | Abcam | Ab126783 |
| GAPDH | Abcam | Ab8245 |
| Ki67 | Abcam | Ab15580 |
| Goat Anti-Rabbit IgG H&L (HRP) | Abcam | Ab6721 |
| Goat Anti-Mouse IgG H&L (HRP) | Abcam | Ab6789 |

**Table S4** The relationship between the expression of SLERCC and various clinicopathological variables in the TCGA database.

| **Characteristics** | **SLERCC expression** | | **P value** |
| --- | --- | --- | --- |
|  | **Low** | **High** |  |
| Total | 269 | 270 |  |
| Age categorized, y |  |  | 0.093 |
| ≤ 60 | 124 (23.0%) | 145 (26.9%) |  |
| > 60 | 145 (26.9%) | 125 (23.2%) |  |
| Gender |  |  | 0.184 |
| Female | 85 (15.8%) | 101 (18.7%) |  |
| Male | 184 (34.1%) | 169 (31.4%) |  |
| T-stage |  |  | **<0.001** |
| T1 | 117 (21.7%) | 161 (29.9%) |  |
| T2 | 35 (6.5%) | 36 (6.7%) |  |
| T3 | 109 (20.2%) | 70 (13.0%) |  |
| T4 | 8 (1.5%) | 3 (0.6%) |  |
| N-stage |  |  | 0.429 |
| N0 | 118 (21.9%) | 123 (22.8%) |  |
| N1 | 10 (1.9%) | 6 (1.1%) |  |
| NX | 141 (26.2%) | 141 (26.2%) |  |
| M-stage |  |  | **0.033** |
| M0 | 204 (37.8%) | 224 (41.6%) |  |
| M1 | 48 (8.9%) | 30 (5.6%) |  |
| MX | 17 (3.2%) | 16 (3.0%) |  |
| Pathologic stage |  |  | **<0.001** |
| Stage I | 114 (21.2%) | 158 (29.3%) |  |
| Stage II | 27 (5.0%) | 32 (5.9%) |  |
| Stage III | 75 (13.9%) | 48 (8.9%) |  |
| Stage IV | 52 (9.6%) | 30 (5.6%) |  |
| Unknown | 1 (0.2%) | 2 (0.4%) |  |
| Histologic grade |  |  | **<0.001** |
| Grade I | 5 (0.9%) | 9 (1.7%) |  |
| Grade II | 98 (18.2%) | 137 (25.4%) |  |
| Grade III | 105 (19.5%) | 102 (18.9%) |  |
| Grade IV | 58 (10.8%) | 17 (3.2%) |  |
| Unknown | 3 (0.6%) | 5 (0.9%) |  |

**Table S5** Univariate and multivariate analyses of factors associated with overall survival (OS) in the TCGA database.

| **Characteristics** | **Total** | **Univariate analysis** | | **Multivariate analysis** | |
| --- | --- | --- | --- | --- | --- |
|  |  | **Hazard ratio (95% CI)** | **P value** | **Hazard ratio (95% CI)** | **P value** |
| Age categorized, y | 539 |  |  |  |  |
| ≤ 60 | 269 | Reference |  | Reference |  |
| > 60 | 270 | 1.755 (1.286-2.396) | **<0.001** | 1.716 (0.946-2.639) | 0.074 |
| Gender | 539 |  |  |  |  |
| Female | 186 | Reference |  | Reference |  |
| Male | 353 | 1.094 (0.794-1.508) | 0.583 | 1.043 (0.672-1.619) | 0.850 |
| T-stage | 539 |  |  |  |  |
| T1/T2 | 349 | Reference |  | Reference |  |
| T3/T4 | 190 | 1.936 (1.634-2.295) | **<0.001** | 1.556 (0.685-3.532) | 0.291 |
| N-stage | 257 |  |  |  |  |
| N0 | 241 | Reference |  | Reference |  |
| N1 | 16 | 2.834 (1.465-5.484) | **0.002** | 1.923 (0.960-3.850) | 0.065 |
| M-stage | 506 |  |  |  |  |
| M0 | 428 | Reference |  | Reference |  |
| M1 | 78 | 4.279 (3.097-5.913) | **<0.001** | 3.028 (1.903-4.819) | **<0.001** |
| Pathologic stage | 536 |  |  |  |  |
| Stage I/II | 331 | Reference |  | Reference |  |
| Stage III/IV | 205 | 3.946 (2.872-5.423) | **<0.001** | 1.154 (0.453-2.938) | 0.763 |
| Histologic grade | 531 |  |  |  |  |
| Grade I/II | 249 | Reference |  | Reference |  |
| Grade III/IV | 282 | 2.357 (1.903-2.919) | **<0.001** | 1.795 (1.337-2.411) | **<0.001** |
| SLERCC | 539 |  |  |  |  |
| Low | 269 | Reference |  | Reference |  |
| High | 270 | 0.515 (0.336-0.790) | **0.002** | 0.544 (0.353-0.839) | **0.006** |

**Table S6** Univariate and multivariate analyses of factors associated with disease free survival (DFS) in the TCGA database.

| **Characteristics** | **Total** | **Univariate analysis** | | **Multivariate analysis** | |
| --- | --- | --- | --- | --- | --- |
|  |  | **Hazard ratio (95% CI)** | **P value** | **Hazard ratio (95% CI)** | **P value** |
| Age categorized, y | 528 |  |  |  |  |
| ≤ 60 | 265 | Reference |  | Reference |  |
| > 60 | 263 | 1.335 (0.914-1.950) | 0.135 | 1.396 (0.812-2.403) | 0.228 |
| Gender | 528 |  |  |  |  |
| Female | 180 | Reference |  | Reference |  |
| Male | 348 | 1.220 (0.807-1.845) | 0.346 | 1.033 (0.594-1.799) | 0.908 |
| T-stage | 528 |  |  |  |  |
| T1/T2 | 346 | Reference |  | Reference |  |
| T3/T4 | 181 | 5.542 (3.652-8.411) | **<0.001** | 1.296 (0.561-2.998) | 0.544 |
| N-stage | 255 |  |  |  |  |
| N0 | 240 | Reference |  | Reference |  |
| N1 | 15 | 3.852 (1.825-8.132) | **<0.001** | 1.633 (0.756-3.525) | 0.212 |
| M-stage | 495 |  |  |  |  |
| M0 | 421 | Reference |  | Reference |  |
| M1 | 74 | 9.108 (6.209-13.361) | **<0.001** | 2.114 (1.089-4.104) | **0.027** |
| Pathologic stage | 525 |  |  |  |  |
| Stage I/II | 328 | Reference |  | Reference |  |
| Stage III/IV | 197 | 9.835 (5.925-16.325) | **<0.001** | 2.737 (0.910-8.237) | 0.073 |
| Histologic grade | 520 |  |  |  |  |
| Grade I/II | 248 | Reference |  | Reference |  |
| Grade III/IV | 271 | 4.793 (2.889-7.952) | **<0.001** | 2.114 (1.089-4.104) | **0.027** |
| SLERCC | 528 |  |  |  |  |
| Low | 263 | Reference |  | Reference |  |
| High | 265 | 0.361 (0.239-0.546) | **<0.001** | 0.526 (0.309-0.893) | **0.017** |
